# Same same, but different: A psychometric examination of three frequently used experimental tasks for cognitive bias assessment in a sample of healthy young adults

**DOI:** 10.3758/s13428-022-01804-9

**Published:** 2022-06-01

**Authors:** Alla Machulska, Kristian Kleinke, Tim Klucken

**Affiliations:** 1 Department of Clinical Psychology, University of Siegen, Siegen, Germany; 2 Department of Psychology, Adolf-Reichwein-Str. 2a, D–57068 Siegen, Siegen, Germany

**Keywords:** Cognitive bias, Information processing, Experimental tasks, Psychometric properties, Reliability, Validity

## Abstract

Cognitive bias research draws upon the notion that altered information processing is key for understanding psychological functioning and well-being. However, little attention has been paid to the question of whether the frequently used experimental paradigms hold adequate psychometric properties. The present study examined the psychometric properties of three widely used cognitive bias tasks: the Approach-Avoidance Task (AAT), the visual dot-probe-task, and the Implicit Association Test (IAT). Approach, attentional, and association biases towards valenced stimuli were repeatedly measured at five different time points in a sample of 79 healthy young adults. Two different devices were used for assessment: a personal computer (PC) and a touchscreen-based tablet. Reliability estimates included internal consistency and temporal stability. Validity was inferred from convergence across different behavioral tasks and correlations between bias scores and self-reported psychological traits. Reliability ranged widely amongst tasks, assessment devices, and measurement time points. While the dot-probe-task appeared to be completely unreliable, bias scores obtained from the PC-based version of the AAT and both (PC and touchscreen) versions of the IAT showed moderate reliability. Almost no associations were found across information processing tasks or between implicit and explicit measures. Cognitive bias research should adopt a standard practice to routinely estimate and report psychometric properties of experimental paradigms, investigate feasible ways to develop more reliable tools, and use tasks that are suitable to answer the precise research question asked.

**Supplementary Information:**

The online version contains supplementary material available at 10.3758/s13428-022-01804-9.

The way people perceive and interpret emotional information is highly subjective and depends on interindividual predispositions, past experiences, and contextual factors. As a result, the same situation can evoke different interpretations. Moreover, most of the time people are confronted with a wide range of information, including positive, neutral, ambiguous, or negative cues. Again, the tendency to preferentially process one sort of information (i.e., more positive or more negative cues) varies from individual to individual. A common notion in prominent cognitive models is that the preferential processing of positive information might not only be explained evolutionarily (Phaf et al., 2014), but also serves as a resilience factor for psychological well-being (Paulus & Wentura, 2013). For example, early studies on embodied cognition show that people tend to automatically approach positive cues and avoid negative or threatening ones (Solarz, 1960), indicating a reciprocal link between evaluation and action (Laham et al., 2014). Moreover, it has been shown that attending to positive information might constitute an emotion regulation strategy, which in turn can serve as a resilience factor to maintain well-being during difficult stages of life (Demeyer et al., 2017). This positive information processing bias has been shown for automatic approach-avoidance tendencies (Becker et al., 2015), attention allocation (Joormann & Gotlib, 2007), and automatic associations (Leppänen, 2006). Interestingly, evidence hints to the fact that those biases are not content-specific, but represent a general orientation (Broeren & Lester, 2013). When it comes to psychopathology, this protective factor seems to be absent or even converted into negative information processing biases or the so-called “cognitive biases.” In recent years, such biases have been a central focus of both clinical psychological theorizing and research. For one part, taking information processing into account can improve our understanding of the development and maintenance of emotional disorders. Beyond that, biased information processing has important implications for treatment, meaning that effective treatment interventions should target those biases in terms of reducing negative processing biases and fostering positive processing (Craske & Pontillo, 2001). Indeed, the last two decades experienced a vast development of a rather new research area aiming at directly modifying cognitive biases in psychopathology (i.e., Cognitive Bias Modification, CBM; for a review, see Fodor et al., 2020). Investigating those biases, however—either in terms of symptom assessment or as a target of intervention—calls for specific task requirements: Measurement procedures for cognitive biases should be reliable, temporally stable, independent of measurement device, and relate in some degree to the underlying process in question and self-reported or observable behavior. Although psychological research has witnessed an array of experimental approaches and novel tasks designed for cognitive bias assessment, little is known as to whether those tasks truly measure the concept of interest and/or to what extent those tasks are contaminated by measurement error. What is even more worrisome is that most cognitive bias assessment and modification studies fail to report psychometric properties at all (Parsons et al., 2018). The present study aims at bridging this gap by systematically investigating psychometric properties of three frequently used experimental tasks for approach, attentional, and association bias assessment: the Approach-Avoidance Task (AAT; Rinck & Becker, 2007), the visual dot-probe task (Miller & Fillmore, 2010), and the Implicit Association Test (IAT; Greenwald et al., 1998), respectively.

These tasks have been commonly used in the context of psychological well-being (i.e., Pool et al., 2016) as well as in psychopathology, including emotional disorders (Hofmann et al., 2008) and substance use (Rooke et al., 2008). However, findings have not always been consistent. For example, some studies either failed to observe group differences (Cisler et al., 2009; Kruijt et al., 2018; Woud et al., 2016) or were unable to find bias change following CBM interventions (Kakoschke et al., 2017). Reasons for mixed results may lie in differing degrees of correspondence between implicit measures and behaviors and/or in highly heterogeneous study designs in terms of trial number, stimulus sets, duration of stimulus presentation, measurement device, or sample characteristics. The former issue refers to the “correspondence principle” (Ajzen & Fishbein, 1977), which posits that measures are a better predictor of criteria (i.e., a particular behavior) when both are conceptualized on a comparable level of generality or specificity (Irving & Smith, 2020). Hence, with regards to trial design and study planning, experimental tasks and dependent outcomes should be selected with close scrutiny. Heterogeneity in task designs, on the other hand, can have profound effects on psychometric properties, and undermines comparability across studies. Furthermore, when it comes to sample characteristics, heterogeneity in terms of degree of between-subject variability can actually have counterintuitive or even paradoxical effects on reliability (see Hedge et al., 2018, for the so-called “reliability paradox”). That is, low reliability for individual differences can emerge from low variance between individuals (i.e., homogeneous samples) when measurement error is held constant across conditions (i.e., between sessions). This renders it essential to systematically investigate and report psychometric properties associated with those tasks, as reliability estimates can vary considerably depending on the particular study design. Moreover, the ubiquitous application of cognitive bias paradigms requires high measurement accuracy and temporal stability for findings to be credible. For instance, changes in biases should be clearly attributed to changes in information processing instead of measurement error, noise, or random fluctuations. However, most studies on cognitive biases fail to routinely report psychometric properties (Parsons et al., 2018). Those that do, frequently report low reliability estimates (Ataya et al., 2012; Schmukle, 2005; Staugaard, 2009). This seems to be particularly true for the dot-probe task, since many studies have shown that attention bias scores derived from the task are characterized by poor internal consistency (Chapman et al., 2019; Kappenman et al., 2014; Vervoort et al., 2021; Waechter et al., 2013), as well as poor test-retest reliability (Brown et al., 2014; Molloy & Anderson, 2020; Schmukle, 2005; Staugaard, 2009). Specifically, reliability indices tend to be not significantly different from zero. Somewhat better results have been reported for the AAT, with internal reliability and test-retest reliability commonly varying between .35 and .77 (Reddy et al., 2016; Reinecke et al., 2010; Zech et al., 2020). However, conflicting results have also been observed. For example, while Kersbergen and colleagues (2015) provide evidence that task instructions might contribute to reliability, this could not be confirmed by other researchers (Reinecke et al., 2010). In addition, some studies report both poor internal reliability (Paulus et al., 2017) and temporal stability (Brown et al., 2014), indicating an overall heterogeneity when examining psychometric properties associated with the AAT. Regarding the IAT, overall satisfactory and/or good internal consistencies have been reported, with split-half correlations and Cronbach’s alpha ranging between 0.60 and 0.90 (Cunningham et al., 2001; Karpinski & Steinman, 2006; Nosek, 2005, Nosek et al., 2007). In line with this, a recent meta-analytic approach (Greenwald & Lai, 2020) yielded an overall Cronbach’s alpha of .77. Test-retest reliability, however, tends to be significantly lower. The same meta-analysis, for example, reported a test-retest correlation of .44 (Greenwald & Lai, 2020). Although higher estimates of test-retest reliability have also been reported (i.e., Egloff et al., 2005), there seems to be a profound variation in the size of temporal stability estimates (for a review, see Lane et al., 2007). Overall, temporal stability of behavioral tasks is substantially lower than for self-report measures (Enkavi et al., 2019).

Taken together, literature on psychometric properties of indirect tasks points to a high degree of heterogeneity. Moreover, although there have been several reviews of psychometric properties, systematic investigations incorporating different measures of reliability and comparing different experimental tasks are missing (for an exception in the context of biases toward thread in children, see Brown et al., 2014). On a related matter, there is considerable variation regarding the precise computer devices used to apply cognitive bias tasks (i.e., PCs, notebooks, tablets, smartphones, etc.). Given the rising popularity and availability of touchscreen-based devices, cognitive bias assessment would profit from using such approaches, and some authors have already undertaken attempts to implement cognitive bias tasks on touchscreen monitors (Meule et al., 2019). However, it remains elusive whether measurements derived from different devices are comparable. Thus, there is a high need for systematically examining the extent to which bias indices from these tasks are reliable, stable over time, and are comparable across different assessment tools (i.e., PC- vs. touchscreen-based assessment).

In addition to the requirement that experimental paradigms should be reliable, information-processing tasks should be a valid measure of implicit cognition and behavior. In this instance, theoretical frameworks in psychopathology assume that cognitive biases do not operate independently from each another, but instead influence one other in a reciprocal manner (see “the combined cognitive bias hypothesis” as proposed by Hirsch et al., 2006). Thus, an important issue regarding cognitive biases concerns the pattern of associations between different measurement tasks.

Finally, the idea that cognitive biases contribute to psychological well-being vs. pathology also means that bias indices derived from indirect tasks should relate to actual or self-reported behavior. That means that attending to positive information should mimic positive well-being while biases toward negative or pathology-related stimuli should be associated with emotional disorders and/or symptoms. While some research suggests that implicit cognition is a reliable predictor of behavior (Paulus et al., 2017; Rooke et al., 2008), others failed to report a link between implicit and explicit cognition or implicit cognition and behavior (Brown et al., 2014; Hagan et al., 2020; Kappenman et al., 2014; Kruijt et al., 2018; Vervoort et al., 2021). It appears that most systematic investigations on the relationship between implicit measures and behavior have been conducted for the IAT (Greenwald et al., 2009; Hofmann et al., 2005; Schmukle & Egloff, 2004). For instance, a recent meta-analysis using data from 217 research reports (Kurdi et al., 2019) found evidence for small, but consistent associations between the IAT and behavior. Most importantly, several moderators have been identified, indicating that associations differed greatly as a function of methodological features. Amongst other variables, high implicit-criterion correspondence produced significantly larger correlations with behavior. Apart from the IAT, systematic investigations of associations between cognitive bias measures and actual behavior have been scarce and mostly inconsistent.

The present study examined the psychometric properties of the AAT, the visual dot-probe task, and the IAT. To do so, within-task and temporal stability were calculated by using split-half and test-retest estimates of reliability. In addition, we compared results drawn from two different measurement devices: PC- vs. touchscreen-based bias assessment. As this study is not placed within a disorder-specific context, but includes a sample of healthy adults, general information processing biases for positive stimuli as compared to negative stimuli were measured. Validity was examined by correlating bias scores derived from each task with one another and by correlating each bias score with self-report data (i.e., psychological well-being and associated trait and personality factors).

## Materials and methods

### Participants

**Fig. 1:**
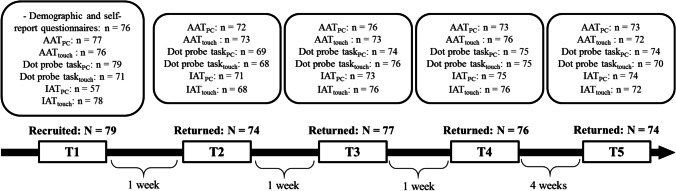
Participants’ time flow**.** AAT = Approach-Avoidance Task; IAT = Implicit Association Task; T = time point; for each task and time point, n represents the number of participants included in the final analyses. Reasons for excluded observations comprise technical or coding errors

A total of 79 adult participants (18 male) were included in the study, all of whom were first-semester students from the University of Siegen (Germany). Participants’ mean age was 21.19 (standard deviation: 3.57). Exclusion criteria for all participants were a history of major medical or psychiatric disorders, insufficient German language skills, or uncorrected visual or auditory impairment. Participants took part in five assessment points. The mean test-retest interval for the first four time points was 1 week, whereas the last assessment was scheduled 4 weeks after the fourth. Figure 1 shows the time flow and response rate for the study. As can be seen, attrition was extremely low. Only two participants terminated their participation after the first assessment point and did not appear at any other time point. Subjects received either money (10 euro/h) or course credit for participation.

Power analyses were based on our primary objective to investigate whether the commonly used experimental tasks are reliable tools for cognitive bias assessment. Building on previous literature and concurring suggestions for suitable reliability, power analyses using G*Power 3.1 (open-source software; Faul et al., 2009) indicated that our sample size had more than adequate power (1 − β = .997, at an alpha α = .05) to detect a moderate effect size (*r* ≥ .5; Koo & Li, 2016). It has to be noted, however, that the expected effect size for implicit–explicit correlations is much lower, ranging around *r* = .20. Power analyses indicated that based on the current sample size, power was inadequate to detect such small associations (1 − β = .427, at an alpha α = .05). Therefore, reported analyses on the relationship between implicit bias scores and self-report measures are exploratory and should be interpreted with caution.

### Ethics statement

The study protocol was approved by the local Ethics Committee of the University of Siegen and was conducted in accordance with the Declaration of Helsinki and Good Clinical Practice guidelines. Participation was voluntary and participants had the right to withdraw their consent for participation at any time.

### Experimental paradigms

All tasks were programmed and operated in Inquisit 5 Lab software, apart from the PC version of the AAT, which was programmed in Microsoft Visual Basic. Experimental paradigms were presented once in a PC-based and once in a touchscreen-based version. For the PC-based task presentation, a desktop computer (Dell Version 1903) was used, to which a 23.8-inch computer monitor (Dell E2216H) with a resolution of 1920 × 1080 pixels was attached. Touchscreen-based tasks were displayed on a Microsoft Surface Pro 8 tablet with a 10.6-inch display and a resolution of 1920 × 1080 pixels.

#### Approach-Avoidance Task (AAT)

Positive and negative pictures (50 for each category) were extracted from the Geneva Affective Picture Database (GAPED; Dan-Glauser & Scherer, 2010). Pictures depicted humans, animals, and nature shots and were thoroughly validated for valence and normative significance previously. Each trial started with a picture that appeared in the center of the computer screen (PC version) or the touchscreen (touch version). Pictures were slightly (3°) tilted to the left or the right and participants had to pull images rotated to the left and to push images rotated to the right. Thus, an indirect task instruction was employed. In the PC version of the task, pull and push movements were executed by means of a joystick (Logitech Extreme 3D) attached to the computer, whereas a wiping gesture (down for pull, up for push) allowed for approach and avoidance movements in the touch version. Upon a pull movement, images grew in size, whereas upon a push movement, images shrank, creating a sense of visually approaching or avoiding the stimuli. Images remained on the screen until the correct full movement was displayed. Trials started with 12 practice trials which comprised neutral images. Thereafter, each picture was shown once in push-away format and once in pull-closer format, resulting in 200 assessment trials. Prior to calculating bias scores, error trials were removed. In accordance with previous work (Rinck & Becker, 2007) an approach bias score was calculated by subtracting median reaction times (RT) for pulling a picture from median RTs for pushing the exact same picture. The reaction time was defined as the time in milliseconds (ms) a participant needed to execute the correct full movement. Accordingly, a positive value indicates an approach tendency toward a picture category, whereas a negative value indicates an avoidance tendency.

#### Visual dot-probe task

As with the AAT, positive and negative pictures (40 per category) were derived from the GAPED. To avoid training effects, all pictures were different from those used in the AAT. After presentation of a fixation cross in the center of the screen (500 ms), a positive and a negative picture appeared side by side on the left and right side of the screen, 3 cm apart. The position of the pictures was randomly chosen to be either left or right to the location of the fixation cross. After a short duration of 1000 ms, the two pictures disappeared and a probe stimulus (here: X) appeared in the location of one of the pictures. In the PC version of the task, participants were asked to indicate probe location via a response pad button press (Cedrus Response Pad RB844). Response pads were used to avoid accuracy problems that have been previously associated with standard PC keyboards (Plant & Turner, 2009). In the touch version, participants touched a marked screen area to indicate whether the probe was left or right. Each image pair was presented once, resulting in 40 test trials. Following Miller and Fillmore (2010) and to minimize habituation effects, 40 filler trials consisting of 10 pairs of neutral images were also included, but not used for the final data analysis. Prior to calculating the bias score, error trials were removed. To calculate an attention bias score, median RTs for probes replacing positive pictures were subtracted from median RTs for probes replacing negative pictures (see Becker et al., 2015). Thus, a positive value mirrors an attention bias towards positive information, while a negative score indicates an attention bias in favor of negative pictures.

#### Implicit Association Test (IAT)

To assess a set of near-universal implicit associations, the target items were chosen to represent flowers vs. insects (see Greenwald et al., 1998). During the task, positive or negative attributes (eight per category; e.g., the German words for "joyful"; "ugly") and target items (eight per category; e.g., images of a "daisy" vs. a "wasp") appeared on the center of the screen. Target items were selected from the Millisecond library script (Millisecond Software, 2015). Participants were asked to categorize positive and negative attributes and target items into predetermined categories via response pad button presses (PC version) or touching a marked screen area (touch version). In line with the literature (Lane et al., 2007), the IAT was organized in seven blocks: (a) a 20-trial target discrimination block (e.g., press yellow for “Flower” vs. press green for “Insect”); (b) a 20-trial attribute discrimination block (e.g., yellow for “Good” vs. green for “Bad”); (c) a 20-trial practice combined block (e.g., yellow for “Flower” OR “Good” vs. green for “Insect” OR “Bad”); (d) a 40-trial test combined block (same as practice); (e) a 20-trial target discrimination block, in which the target categories were reversed (e.g., yellow for “Insect” vs. green for “Flower”); (f) a 20-trial practice combined block with reversed target categories (e.g., yellow for “Insect” OR “Good” vs. green for “Flower” OR “Bad”), and (g) a 40-trial test combined block (same as practice). Blocks c, d, f, and g were crucial blocks used in scoring the IAT. To prevent methodological compounds, target position and block order were counterbalanced. Trials in which flowers and positive attributes (or insects and negative attributes) shared a response key were supposed to be congruent and vice versa. Congruent and incongruent blocks comprised 60 trials each (20-trial practice combined block + 40-trial test combined block), resulting in 120 trials included in the analysis. Error trials were handled by requiring respondents to correct their responses. Hence, no replacement procedure for incorrect responses was required (Greenwald et al., 2003; Lane et al., 2007). Following recommendations by Greenwald et al. (2003), subjects for whom more than 10% of trials had latencies of less than 300 ms were excluded from further analyses. In addition, trials with above 10,000 ms were eliminated. The IAT bias score was calculated using the improved scoring algorithm (D-score) as recommended by Greenwald et al. (2003): Mean RTs for congruent blocks were subtracted from mean RTs for incongruent blocks; this difference score was standardized by dividing individuals' response time differences by a personalized standard deviation of these response latencies. Larger IAT D-scores suggest stronger implicit, positive associations with flowers.

### Self-report measures

During the first assessment session, participants completed an extensive set of questionnaire measures, including general well-being, personality traits, and other traits implicated in psychological functioning. Self-report measures comprised (1) Positive Mental Health (PMH; Lukat et al., 2016); (2) Neuroticism-Extraversion-Openness–Five-Factor Inventory (NEO-FFI) for measuring the Big Five personality structure (Costa Jr & McCrae, 1985; German version: Borkenau & Ostendorf, 2008); (3) Affective Neuroscience Personality Scales (ANPS; German version: Reuter et al., 2017); (4) State-Trait Anxiety Inventory (STAI; German version: Laux et al., 1981); (5) Anxiety Sensitivity Index (ASI-4; German version: Kemper et al., 2010); (6) Anxiety Coping Index (ABI; German version: Krohne & Egloff, 1999); (7) Disgust Scale (German version: Haidt et al., 1994); and (8) Need Inventory of Sensation Seeking (NISS; German version: Roth et al., 2014). Questionnaires were presented in paper-pencil format.

### Procedure

The study consisted of five data collection time points (see Fig. 1). Each time point was one week apart, with the exception of the last time point (*t*_*5*_) which took place four weeks after *t*_*4*_. Questionnaire data was only administered at *t*_*1*_, and experimental paradigms for cognitive bias measurement (AAT-PC, AAT-touch, dot-probe-PC, dot-probe-touch, IAT-PC, IAT-touch) were assessed at each time point. Hence, each participant completed 30 tasks in total. The order of experimental tasks was counterbalanced across participants using a Latin square design. The individual’s task order was identical for all assessment points. Each testing took place in groups of up to four participants and lasted for about 60 minutes. To prevent fatigue and/or exhaustion, participants were allowed to take a break as required.

### Planned analyses and missing data handling

Internal consistency of the measurements was quantified using the split-half-method. More precisely, reliability estimates for bias scores derived from the AAT and the dot-probe task were determined by means of correlations between the odd and even trial numbers respectively. Internal consistency for D-scores derived from the IAT was calculated by correlating the first (practice) and the second (test) block as recommended by Greenwald et al. (2003). Stability across time of the respective bias scores was inferred from their bivariate autocorrelations; 95% confidence intervals for the correlation coefficients can be obtained from the supplemental material appendix. Interrelationships between the assessment device of the respective bias scores (i.e., PC-based versus touchscreen-based assessment), as well as relationships between the bias scores regarding their stability across time, were analyzed by bivariate or multivariate autoregressive models (ARM; Jöreskog, 1979). The autoregressive coefficients in ARM express the individuals’ relative stability (i.e., rank order stability) in the variable of interest.

Please note that we applied existing recommendations and most common approaches to calculate bias scores for each task (see “Experimental paradigms” subsection). That is, the D-score algorithm was used to calculate association biases derived from the IAT, while differences between median RTs were used to calculate approach-avoidance (AAT) and attentional biases (dot-probe task). This approach was chosen to increase comparability with existing studies. However, we understand that by doing so, within-task comparability is limited. Therefore, we ran additional analyses in which the exact same formula was applied to each task (either the D-score algorithm or the median difference for all three tasks). We added these extended analyses to the supplemental material appendix.

Three cases were identified which were characterized by extreme high error rates (average error rate ≥ 20%) throughout most time points and experimental tasks. Therefore, those cases were excluded from further reliability and validity analyses.

Loss of information due to missing data was either compensated for by full information maximum likelihood (FIML) estimation or by multiple imputation (MI). For all analyses, missing data were assumed to be missing at random in the sense of Rubin (1976). For all analyses that involved bivariate correlations, missing data were multiply imputed (*m* = 100 times) by the R package mice (van Buuren & Groothuis-Oudshoorn, 2011) using an iterative predictive mean matching approach with distance-based donor selection, proposed by Siddique and Belin (2008) and implemented into the mice framework by Gaffert et al. (2016). We ran 20 iterations of mice’s Gibbs sampler respectively and assessed convergence by graphical inspection of the trace line plots of the respective estimated parameter value against the iteration number. For a detailed discussion of these procedures, see Kleinke et al. (2020). Combined estimates of the respective correlation coefficients were obtained using the approach outlined in Schafer (1997). Here, the normal theory MI combination rules are applied to the Fisher-z-transformed correlation coefficients. After the MI pooling was done, correlations were then back-transformed to the original scale. For the ARM, we simply obtained FIML estimates, which is usually the missing data method of choice for more complex path or structural equation models.

## Results

### Self-report measures

Descriptive variables and self-report measures concerning positive well-being, personality traits, and trait anxiety are presented in Table 1. Overall, mean scores and standard deviations were comparable to those reported for healthy young samples.

**Table 1:** Descriptive statistic of demographic variables, personality traits, and mental health variables

Variable	*N*	M (SD)	Range	Cronbach’s α
Age (years)	77	21.19 (3.57)	18–39	-
Gender (% female)	78	77	-	-
NEO-FFI				
Openness to experience	76	32.99 (6.48)	17–47	.74
Conscientiousness	76	33.21 (7.61)	12–46	.87
Extraversion	76	28.22 (7.07)	10–42	.83
Agreeableness	76	33.27 (6.41)	11–44	.80
Neuroticism	76	21.43 (7.77)	3–42	.85
ANPS				
SEEKING	76	40.25 (4.07)	30–49	.61
PLAY	76	41.28 (5.64)	26–52	.77
CARE	76	42.51 (5.20)	28–53	.71
FEAR	76	37.08 (6.94)	24–52	.87
ANGER	76	35.23 (7.06)	19–53	.87
SADNESS	76	34.26 (4.87)	25–46	.68
Spirituality	76	28.68 (7.71)	12–46	.90
Lie	76	10.88 (2.12)	6–17	.56
Positive mental health (PMH)	75	20.00 (4.46)	8–27	.87
Anxiety				
STAI-G	75	40.82 (9.26)	26–67	.90
ASI-4	73	31.64 (15.04)	7–90	.91
ABI: Vigilance	76	23.29 (7.34)	5–36	.87
ABI: Cognitive avoidance	76	22.55 (6.63)	5–37	.84
Sensation seeking				
NISS: Need for stimulation	76	2.92 (.69)	1.55–4.64	.88
NISS: Avoidance of rest	76	2.50 (.82)	1.00–4.33	.82
NISS: Sum score	76	2.77 (.56)	1.53–3.82	.84
Disgust Scale	73	17.06 (5.03)	8.70–28	.83

*NEO-FFI* Neuroticism-Extraversion-Openness–Five-Factor Inventory, *ANPS* Affective Personality Scales, *STAI-G* State-Trait Anxiety Inventory German version, *ASI-4* Anxiety Sensitivity Index 4, *ABI* Anxiety Coping Index, *NISS* Need Inventory of Sensation Seeking, *N* number of observed cases, *M* mean, *SD* standard deviation

### Internal consistency: Split-half correlations

#### AAT

In the PC version of the task, split-half correlations regarding the approach bias were moderate to good and ranged between *r* = 0.45 (Spearman-Brown-corrected *r*_sb_ = .62) and *r* = 0.60 (*r*_sb_ = .75) for negative pictures, and between *r* = 0.43 (*r*_*sb*_ = .60) and *r* = 0.63 (*r*_sb_ = .77) for positive pictures (for detailed results, see Table 2). When assessed via touchscreen, results were largely comparable, but correlations between odd and even trials were near zero for negative pictures at *t*_*2*_ and for positive pictures at *t*_*4*_ and *t*_*5*_. Regarding the other time points, split-half reliability coefficients for negative pictures ranged between *r* = 0.35 (*r*_sb_ = .52) and *r* = 0.58 (*r*_sb_ =.73), and between *r* = 0.29 (*r*_sb_ = .45) and *r* = 0.57 (*r*_sb_ = .73) for positive pictures.

**Table 2:** Internal consistency (split-half correlation) and descriptive statistics for cognitive bias assessment tasks

Task	Device					1. Half		2. Half	
		*Time*	*r*	*r*_*SB*_	*n*	*M*	*SD*	*M*	*SD*
*AAT*									
Negative approach bias	PC	1	0.55^***^	0.71^***^	74	4.50	91.14	−11.95	69.06
		2	0.60^***^	0.75^***^	69	5.00	64.16	−2.34	79.42
		3	0.45^**^	0.62^***^	73	14.81	58.20	2.73	55.77
		4	0.56^***^	0.72^***^	70	7.44	67.97	.46	66.92
		5	0.60^***^	0.75^***^	70	−3.26	64.37	−10.34	53.74
Positive approach bias		1	0.43^***^	0.60^***^	74	−14.25	87.85	5.47	78.02
		2	0.63^***^	0.77^***^	69	7.05	57.54	9.44	72.30
		3	0.63^***^	0.77^***^	73	10.52	60.26	13.78	61.93
		4	0.58^***^	0.73^***^	70	4.89	58.40	6.33	54.49
		5	0.56^***^	0.72^***^	70	0.00	50.79	−1.49	54.28
Negative approach bias	touch-screen	1	0.48^***^	0.65^***^	74	−3.97	331.63	−4.55	229.42
		2	−0.12	−0.21	69	−1.80	248.49	−21.44	231.03
		3	0.35^**^	0.52^***^	70	27.19	161.03	4.45	210.25
		4	0.58^***^	0.73^***^	73	−7.72	146.65	−5.64	155.96
		5	0.58^***^	0.73^***^	69	6.25	147.70	11.16	123.64
Positive approach bias		1	0.29^*^	0.45^***^	74	10.56	197.56	12.15	306.00
		2	0.58^***^	0.73^***^	69	−66.91	358.56	−18.55	468.01
		3	0.57^***^	0.73^***^	70	−12.41	153.49	−11.84	179.13
		4	0.08	0.15	73	8.97	133.41	4.19	134.24
		5	0.18	0.31^**^	69	0.44	117.08	31.44	218.62
*Dot-probe task*									
Attentional bias	PC	1	−0.18	−0.31^**^	76	9.22	33.84	1.58	29.96
		2	0.16	0.28^*^	67	−1.64	32.54	8.02	28.10
		3	0.08	0.15	71	3.68	28.12	1.94	30.93
		4	0.03	0.06	72	5.94	40.93	−0.47	37.55
		5	−0.30^*^	−0.46^***^	71	6.46	37.87	−2.52	32.00
Attentional bias	touch-screen	1	−0.04	−0.08	68	3.12	33.03	6.03	28.78
		2	−0.11	−0.20	65	4.37	31.81	0.74	30.27
		3	−0.08	−0.15	73	−1.69	24.44	1.35	28.78
		4	0.01	0.02	72	1.75	27.93	9.20	25.32
		5	0.10	0.18	67	3.55	27.88	1.70	32.50
*IAT*									
Association bias (D-score)	PC	1	0.16	-	55	0.62	0.45	0.63	0.37
		2	0.24^*^	-	68	0.58	0.41	0.50	0.39
		3	0.42^***^	-	70	0.58	0.44	0.50	0.40
		4	0.51^***^	-	72	0.37	0.47	0.41	0.39
		5	0.48^***^	-	71	0.34	0.46	0.43	0.36
Association bias (D-score)	touch-screen	1	0.60^***^	-	75	0.57	0.42	0.50	0.41
		2	0.37^**^	-	65	0.51	0.42	0.45	0.44
		3	0.50^***^	-	73	0.43	0.48	0.39	0.37
		4	0.36^**^	-	72	0.31	0.44	0.32	0.40
		5	0.38^**^	-	69	0.26	0.48	0.45	0.35

All bias scores were measured at five different time points. *AAT* Approach-Avoidance Task, *IAT* Implicit Association Task, *r* Pearson’s correlation coefficient, *r*_SB_ Spearman-Brown correction; 1: time point 1; 2: time point 2; 3: time point 3; 4: time point 4; 5: time point 5, *p < .05; **p < .01; ***p < .001, M: mean bias score; SD: standard deviation; *n*: number of observed cases for each task; “*r*” denotes the correlation between the odd and even trials (in the case of AAT and dot-probe test) or the correlation between the first (practice) and second (test) block (in the case of the IAT) respectively and is based on multiple imputation. Due to the fact that internal consistency for the IAT was based in blocks rather than a set of items, a Spearman-Brown correction was not applicable to this case.

#### Dot-probe task

Split-half reliability regarding the attention bias was usually very low. Correlations were mostly not statistically different from zero regardless of the assessment device or even negative (*t*_4; *PC*_ : *r* =  − 0.30, *p* = .009, r_sb_ = −0.46).

#### IAT

Split-half correlations regarding the association bias ranged between *r* = 0.16 and *r* = 0.51 in the PC assessment, and between *r* = 0.36 and *r* = 0.60 when measured via touchscreen^1^.

### Temporal stability: Test-retest reliability

Table 3 displays correlations of the respective bias scores across time.

**Table 3:** Temporal stability (test-retest reliability) for cognitive bias assessment tasks

Task	Device	Test-retest correlations (*r*)				
		Time	*T1*	*T2*	*T3*	*T4*
*AAT*						
Negative approach bias	PC	*T2*	0.44^***^			
		*T3*	0.45^***^	0.54^***^		
		*T4*	0.47^***^	0.48^***^	0.38^***^	
		*T5*	0.43^***^	0.41^***^	0.65^***^	0.54^***^
Positive approach bias		*T2*	0.33^**^			
		*T3*	0.25^*^	0.62^***^		
		*T4*	0.34^**^	0.42^***^	0.38^***^	
		*T5*	0.34^**^	0.41^***^	0.62^***^	0.49^***^
Negative approach bias	Touchscreen	*T2*	−0.05			
		*T3*	0.00	0.21		
		*T4*	0.22	−0.02	0.32^**^	
		*T5*	0.03	0.22	0.24	0.29^*^
Positive approach bias		*T2*	0.07			
		*T3*	0.25^*^	0.00		
		*T4*	0.17	0.15	0.17	
		*T5*	0.12	−0.07	0.29^*^	−0.19
*Dot-probe task*						
Attentional bias	PC	*T2*	−0.14			
		*T3*	−0.20	−0.19		
		*T4*	−0.13	0.08	0.10	
		*T5*	0.09	0.20	0.07	0.12
Attentional bias	Touchscreen	*T2*	0.00			
		*T3*	0.04	−0.08		
		*T4*	0.15	0.10	0.02	
		*T5*	0.03	0.25^*^	−0.09	0.01
*IAT*						
Association bias	PC	*T2*	0.36^**^			
(D-score)		*T3*	0.50^***^	0.35^**^		
		*T4*	0.44^***^	0.38^**^	0.58^***^	
		*T5*	0.36^**^	0.40^***^	0.26^*^	0.31^***^
Association bias	Touchscreen	*T2*	0.54^***^			
(D-score)		*T3*	0.49^***^	0.39^**^		
		*T4*	0.31^**^	0.36^**^	0.51^***^	
		*T5*	0.17	0.25^*^	0.13	0.22

*AAT* Approach-Avoidance Task, *IAT* Implicit Association Task, *RT* reaction time, *r* Pearson’s correlation coefficient, *T1* time point 1, *T2* time point 2, *T3* time point 3, *T4* time point 4, *T5* time point 5, *p < .05; **p < .01; ***p < .001

#### AAT

Approach bias scores for negative or positive pictures were moderately correlated with coefficients ranging between 0.25 and 0.65, but were usually much smaller and—with a few exceptions—nonsignificant when assessed via touchscreen.

#### Dot-probe task

Nearly no autocorrelation regarding the attentional bias was statistically different from zero regardless of the type of assessment (only exception for touchscreen assessment: *r*_*t2t5*_ = .25).

#### IAT

Association bias scores ranged between 0.26 and 0.58 when assessed via PC, and between 0.13 and 0.54 when assessed via touchscreen, and were usually moderate in the majority of cases. Although temporal stability was generally higher when assessed via PC, a time-dependent effect became apparent in the touchscreen-based assessment: correlations between proximal time points tended to be larger than those for distal time points (i.e., *r*_*t1-t2*_ = 0.54, *r*_*t1t3*_ = .49, *r*_*t1t4*_ = .31, *r*_*t1t5*_ = .17).

### Convergence between different assessment devices: PC- vs. touchscreen-based assessment

#### Autoregressive model: AAT negative (PC vs. touch)

**Fig. 2:**
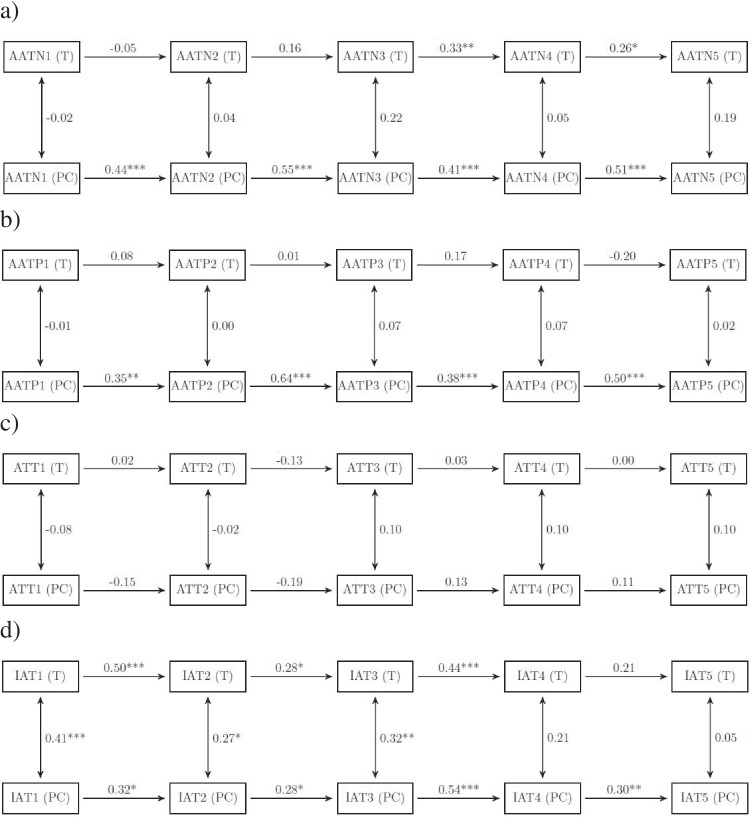
Bivariate autoregressive models comparing the stability of the respective biases of the PC and the touchscreen assessments**.** Panel (**a**) shows the approach-avoidance bias scores for negative cues (AATN: Approach-Avoidance Task-negative), **b** shows approach-avoidance bias scores for positive cues (AATP: Approach-Avoidance Task-positive), **c** displays attentional bias scores (ATT: Attentional Bias), **d** shows IAT bias scores (D-scores; IAT: Implicit Association Task). T and PC denote the touchscreen versus PC assessment devices, respectively. Numbers 1–5 indicate the respective measurement time point. All coefficients are standardized coefficients and were obtained by full information maximum likelihood estimation. *p < .05; **p < .01; ***p < .001

Figure 2a displays a bivariate autoregressive model comparing approach biases for negative cues in the PC versus touchscreen assessments. Bias scores based on PC and touchscreen were usually not correlated, or correlations were only very small (range: −0.02 to 0.22).

#### Autoregressive model: AAT positive (PC vs. touch)

Figure 2b displays the same autoregressive model for approach biases towards positive cues. The general pattern of results is the same as for the negative cues. Bias scores based on PC and touchscreen were not significantly correlated (range: −0.01 to 0.07).

#### Autoregressive model: Attentional bias (PC vs. touch)

Figure 2c displays model results for attentional bias scores. Again, bias scores based on PC and touchscreen assessments were not significantly correlated at each measurement time point (range: −.08 to .10).

#### Autoregressive model: IAT (PC vs. touch)

Finally, Fig. 2d gives model results for association bias scores. At some measurement time points, PC- and touchscreen-based biases were moderately correlated (i.e., for *t*_*1*_ – *t*_*3*_, with correlations ranging between 0.27 and 0.41), only small and nonsignificant correlations could be obtained for the remaining time points.

### Criterion validity: Convergence between cognitive bias measures

**Fig. 3:**
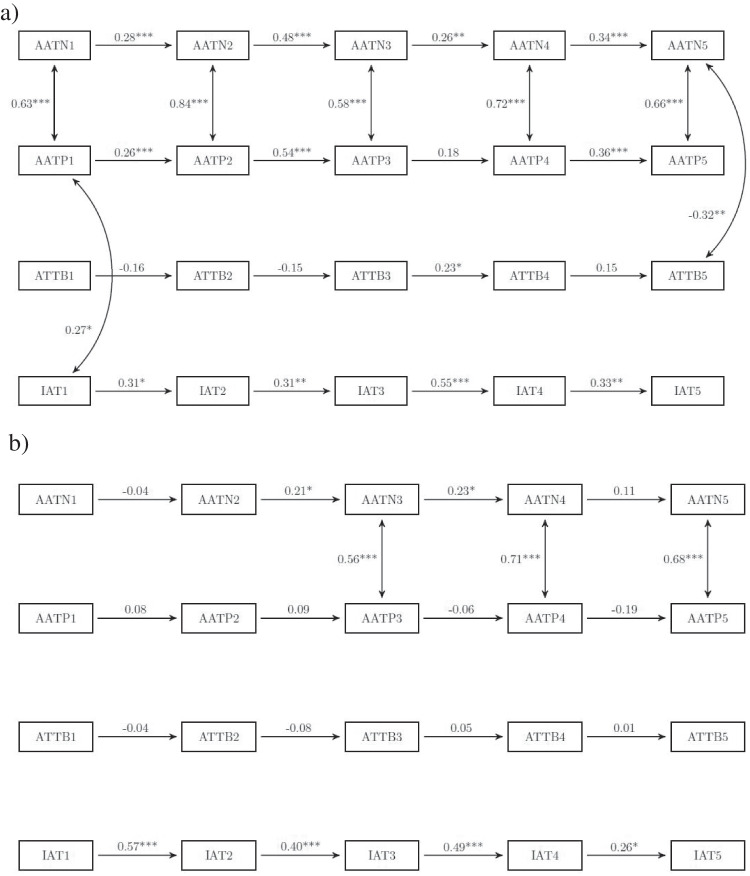
Parallel autoregressive models comparing the stability of the respective biases and their interrelationships at each measurement time point. Panel (**a**) refers to the PC-based assessment; panel (**b**) to the touchscreen-based assessment. AATN and AATP denote bias scores towards negative and positive cues respectively, AAT are attentional bias scores; the last row displays IAT bias scores. Numbers 1–5 indicate the respective measurement time point. All coefficients are standardized coefficients and were obtained by full information maximum likelihood estimation. All regression coefficients are shown. Only the significant correlations are shown. *p < .05; **p < .01; ***p < .001

Figure 3 displays the results of a parallel autoregressive model comprising all bias scores as assessed via PC or touchscreen, respectively.

Of interest are the correlations between different bias scores within (approach biases for positive and negative stimuli measured via the AAT) and across different information-processing tasks (AAT, dot-probe task, and IAT). In the PC assessment, approach biases for positive and negative cues were usually moderately to highly correlated at each measurement time point, with correlations ranging between 0.58 and 0.84.

Interestingly, bias scores across different assessment tasks were mostly not correlated, suggesting that these tasks are measuring different aspects of information processing. For the PC assessment, approach biases for positive cues correlated positively with the IAT-score at *t*_*1*_ (*r* = .27). In addition, approach biases for negative pictures showed an inverse correlation with the attentional bias at *t*_*5*_ (*r* = −.32). No other between-task associations appeared.

In the touchscreen-based assessment, approach biases for positive and negative pictures did not correlate at the first two measurement points, but correlations were high for the remaining time points (range: .56–.71). There was no indication for credible interrelationships between approach, attentional, and association biases.

### Construct validity: Association with self-report measures

Correlations between cognitive biases and self-report measures are presented in Table 4. As can be seen, there were only few significant associations between cognitive biases and personality traits or anxiety. Of interest were the positive correlations between IAT-scores and conscientiousness (for PC: *r* = .38; for touchscreen: *r* = .40). Given the large number of comparisons, however, these correlations should be interpreted with caution, as they may constitute a statistical artefact.

**Table 4:** Pearson’s correlations (*r*) between cognitive biases of the PC and touchscreen assessments at the first measurement time point with self-reported trait variables

Variable	PC assessment				Touchscreen assessment			
	Negative AAT bias	Positive AAT bias	ATT bias	IAT bias	Negative AAT bias	Positive AAT bias	ATT bias	IAT bias
NEO-FFI								
Openness	−0.07	0.08	−0.18	−0.18	0.11	−0.13	−0.03	−0.01
Conscientiousness	0.01	0.21	0.02	0.38^**^	0.09	0.11	−0.05	0.40^***^
Extraversion	−0.08	0.01	−0.06	0.17	0.04	0.10	−0.01	0.06
Agreeableness	−0.10	0.02	−0.14	0.20	0.02	−0.06	0.02	0.04
Neuroticism	−0.06	0.00	−0.03	−0.09	−0.07	−0.09	0.03	0.04
ANPS								
SEEKING	0.02	0.13	−0.04	−0.01	0.06	−0.01	−0.15	0.06
PLAY	−0.15	−0.09	−0.04	0.17	0.08	0.00	−0.01	0.07
CARE	−0.25^*^	−0.07	−0.10	0.01	−0.06	−0.01	−0.05	0.05
FEAR	0.09	0.04	−0.05	−0.09	0.01	0.00	−0.09	0.01
ANGER	0.07	0.08	0.07	0.08	0.03	−0.02	0.02	0.09
SADNESS	0.05	0.13	−0.08	−0.04	0.07	−0.17	0.07	0.00
Spirituality	−0.13	−0.06	−0.19	−0.04	0.07	−0.03	0.02	−0.09
Lie	−0.01	0.01	0.09	0.11	−0.05	0.10	0.00	0.08
Positive mental health (PMH)	0.06	0.05	−0.02	0.12	0.17	0.16	−0.08	0.09
Anxiety								
STAI-G	0.15	0.01	0.02	−0.20	−0.18	−0.06	0.02	−0.18
ASI-4	0.00	−0.12	−0.11	−0.06	−0.09	0.10	−0.08	0.08
ABI: Vigilance	−0.03	−0.15	0.16	0.07	0.01	0.28^*^	−0.18	−0.05
ABI: Cognitive avoidance	0.10	0.19	0.17	0.08	−0.04	0.19	−0.07	0.07
Sensation seeking								
NISS: Need for stimulation	−0.03	−0.08	−0.02	−0.08	−0.07	0.17	−0.11	−0.05
NISS: Avoidance of rest	−0.05	0.03	−0.14	0.17	0.02	0.13	−0.06	−0.06
NISS: Sum score	−0.05	−0.04	−0.09	0.02	−0.04	0.20	−0.11	−0.07
Disgust Scale	0.12	0.14	−0.07	0.01	0.00	0.03	0.03	0.14

*NEO-FFI* Neuroticism-Extraversion-Openness–Five-Factor Inventory, *ANPS* Affective Personality Scales, *STAI-G* State-Trait Anxiety Inventory German version, *ASI-4* Anxiety Sensitivity Index 4, *ABI* Anxiety Coping Index, *NISS* Need Inventory of Sensation Seeking, *AAT* approach/avoidance bias derived from the Approach-Avoidance Task, *ATT bias* attentional bias derived from the dot-probe task, *IAT bias* association bias (D-score) derived from the Implicit Association Test, *p < .05, **p < .01, ***p < .001

## Discussion

The aim of the present study was to systematically examine psychometric properties of three widely used information-processing tasks, namely the AAT, the visual dot-probe task, and the IAT. For this purpose, approach, attentional, and association biases were assessed by means of two different devices (PC and touchscreen) and across multiple time points.

### Reliability

The AAT and IAT showed mostly moderate to good internal consistency and temporal stability (Koo & Li, 2016; Schmukle., 2005). For the PC-AAT, eight (uncorrected) or 10 (after Spearmen-Brown correction) out of 10 split-half reliabilities and five out of 20 test-retest correlations were ≥.50. The touchscreen-AAT showed somewhat poorer reliability estimates, with only four (uncorrected) or six (Spearmen-Brown-corrected) out of 10 split-half reliabilities and none of the test-retest correlations exceeding the .50 margin (Koo & Li, 2016; Schmukle, 2005). Likewise, the IAT showed quite similar internal consistencies and temporal stability independent of measurement device. For one part, our results add to previous literature showing that indirect tasks exhibit somewhat higher internal consistency than test-retest reliability (Connor & Evers, 2020; Greenwald & Lai, 2020). Moreover, despite the time-consuming nature of our study design (i.e., six behavioral tasks per measurement point), fatigue or cognitive exhaustion did not seem to have an impact on internal reliability. Notwithstanding however, to fully explore the impact of extensive and repeated measurements, future studies would benefit from incorporating explicit measures of fatigue.

Most strikingly, the dot-probe task used in our study appeared to be entirely unreliable. In fact, no correlation exceeded the zero-threshold in the sense of statistical significance and one correlation was even significantly negative (or rather two, when applying the Spearmen-Brown correction). This was the case even though we adapted recommendations provided by the literature to increase reliability (Miller & Fillmore, 2010; Townshend & Duka, 2001). Our results are in line with previous findings, which showed that despite the long history of use, reliability estimates of the dot-probe task are unacceptably low (Ataya et al., 2012; Schmukle, 2005; Staugaard, 2009; Vervoort et al., 2021). This suggests that the dot-probe task or at least the crucial measures obtained from the task (i.e., reaction time differences) are not sensitive enough to detect differences in attention allocation.

However, we wish to refer to some recent deliberations in the literature, which emphasize the fact that implicit measures characterized by low reliability do not have to be problematic per se, as long as this is due to low variance between individuals rather than high measurement variance (De Schryver et al., 2016). More specifically, low between-subject variance can stem from homogeneous samples and—if error variance is held constant—can decrease reliability estimates, since reliability in correlational research refers to the extent to which a measure reliably ranks different individuals (Hedge et al., 2018). On the other hand, high between-subject variability will show higher reliability, unless measurement noise increases (De Schryver et al., 2016). With respect to the present study, we need to acknowledge that we included a student sample, which might show reduced variance compared to the general population. At the same time, the sample we used is typical for most social and cognitive studies, yielding high comparability to related research. In addition, the source of variance (i.e., between-subject variance vs. systematic measurement error variance) is difficult to disentangle.

Taken together, both low task reliability and issues contributing to this may explain some inconsistent findings reported in the literature, since reduced reliability limits observable correlations and might reduce the power to detect significant group differences (Kruijt et al., 2018; Parsons et al., 2018). As a result, some researchers addressed the issue of increasing reliability for the dot-probe task, for instance by complementing reaction time measures with those that account for dynamic processes over time or by eye tracking (Field et al., 2006; Field & Christiansen, 2012; Fu & Pérez-Edgar, 2019; Miller & Fillmore, 2010; Molloy & Anderson, 2020; Rodebaugh et al., 2016).

As with the dot-probe task, several proposals have been made to improve the psychometric properties of the AAT and the IAT, respectively. For instance, Kersbergen et al. (2015) compared four different approach-avoidance tasks in terms of validity and reliability and found that psychometric properties were best when direct task instructions were employed. Field et al.  (2011) reported similar results. When applying indirect instructions, reliability seems to be decreased when the content-irrelevant feature is easy to categorize without active processing of the stimulus content (de Hower et al., 2001). The irrelevant feature used in the present AAT was a 3° tilt to the left or right, which represents a rather challenging categorizing characteristic and might explain why we found mostly satisfying reliability estimates for this precise task. Another issue addresses the optimal method to calculate bias scores. Greenwald et al. (2003) examined candidate algorithms for bias score calculations derived from the IAT and found that the best-performing measure (D-score) incorporates data from the IAT practice trials, uses a metric that is calibrated by each respondent's latency variability, eliminates trials with latencies >10,000 ms and subjects for whom more than 10% of trials have latencies of < 300 ms, and requires participants to correct their errors. We followed the authors’ guidelines in our analyses of the IAT. Please note, however, that despite the wide popularity of the D-score algorithm in IAT literature, some recent accounts have proposed alternative indices based on the probabilistic index (PI) as a candidate effect size measure for analyzing data obtained from the IAT (De Schryver et al., 2018). Using both a Monte Carlo simulation and reanalysis of existing data, the authors showed that the PI was less sensitive to outliers and outperformed D-score algorithms on reliability and validity measures. Although similar rigorous examinations are lacking for other information processing tasks, it appears premature to apply these principles to other tasks without previous examination. For example, in the study by Kersbergen et al. (2015), the relevant-feature AAT (direct task instruction) was only valid when the bias score was based on raw RTs instead of the data aggregation method resembling the D-score calculation. On the other hand, some researchers argue to use average scores instead of difference scores, as the former hold more individual variation and therefore are more reliable (Draheim et al., 2019; see also supplemental material appendix for reliability analyses based on mean RTs). On the downside, average scores hinder interpretability of results, as they are indicative of general processing tendencies rather than preferences in emotional information processing (see Brown et al., 2014).

Although these developments certainly contribute to improvements of task designs and psychometric characteristics, we wish to emphasize the importance of adopting a general research practice of estimating and reporting psychometric properties of tasks used to measure cognitive biases. This emphasis not only refers to tasks, which suffer from low reliability such as the dot-probe task, but to each and every behavioral measure used in cognitive bias research. Although better psychometric characteristics were reported for other attentional bias tasks (Ataya et al., 2012) or other cognitive bias measures, including the AAT (Kersbergen et al., 2015) and the IAT (Hofmann et al., 2005), our results indicate that reliability can vary as a function of measurement time and device. For example, internal consistencies varied between .43 and .63 (Spearman-Brown-corrected: .60–.77) for the PC version of the AAT and between .16 and .51 for the PC version of the IAT. In addition, even though reliability estimates remained equally high when association biases were assessed via touchscreen, this was not the case for approach biases, where internal reliability fell dramatically and showed a much wider range (−.12 to .58; Spearman-Brown-corrected: −.21 to .73). This pattern of results is mirrored in the temporal stability indices and correlations between PC- vs. touchscreen-based measures obtained from the AAT and IAT, respectively. Furthermore, there was little systematic convergence across different measurement devices (PC vs. touchscreen) which aimed at measuring the exact same information processing bias. This might be explained by the specific task demands attributed to touchscreen-based assessment. During the touchscreen-based AAT, for instance, prior to reacting to a stimulus in terms of making a pull (approach) or push (avoidance) movement, participants first had to touch the screen and, in this way, the stimulus. This suggests that each reaction was preceded by an approach movement, thereby potentially compounding bias measures and minimizing observed correlations within (internal consistency) and across tasks (temporal stability, correlations between different devices). In light of the development and increasing use of touchscreen devices, it remains unclear whether measures obtained from such devices are constantly trustworthy and should be examined with more detail.

### Validity

Regarding the question of validity, we found little to no indication of a convergence across different cognitive bias measures or between cognitive biases and self-report. However, we wish to emphasize that our power analysis was designed for reliability analyses mainly (our primary objective). That is, while statistical power was reasonable to detect moderate effect sizes (*r* ≥ .50) that are deemed necessary for a specific task to be reliable, power was insufficient for small effect sizes (.15–.20) that are frequently reported in validity studies (Kurdi et al., 2019). Hence, results concerning across-task associations and implicit–explicit associations should be regarded as exploratory and interpreted with caution, needing replication. That said, positive and negative approach bias scores derived from the AAT were moderately to highly correlated, suggesting a genuine underlying behavioral action tendency. However, when looking at different measurement devices (PC vs. touchscreen) within the same task or at different cognitive processes, there was poor coherence between cognitive biases obtained from different tasks. For one part, this finding supports previous work showing little convergence across different processing biases (Broeren et al., 2011; Brown et al., 2014; Dalgleish et al., 2003; Klein et al., 2017). On the other hand, this lack of convergence is difficult to reconcile with cognitive models of psychopathology and the “combined cognitive bias hypothesis” in particular, which assumes that cognitive biases are interrelated and mutually interact with one another in a reciprocal manner (Everaert et al., 2012; Hirsch et al., 2006). There are several possible explanations for this: First, the lack of convergence could be attributed to poor task reliability as reduced reliability limits observable correlation. On a related manner, Rouder et al. (2019) argue that correlations among different cognitive tasks are difficult to prove because of small individual variation. The authors recommend (among other strategies) increasing the number of trials, as a low number of trials is a common source for trial noise and attenuation. Using a calculation example, they conclude that more than 600 trials per condition would be needed to detect a 25 ms true individual variability. Although such high numbers of trials are likely to be unfeasible for most study protocols, it appears that more trials are better (Enkavi et al., 2019). For example, Hedge et al. (2018) found that reliability of many reaction time-based effects plateaued after approximately 100 trials per condition. While the AAT used in the current study meet this requirement, the IAT comprised 60 trials per condition and the dot-probe task included only 40 test trials. This was done to comply with the most frequent task adaptations from the literature (Miller & Fillmore, 2010); however, it is reasonable to assume that both tasks could be improved by increasing the trial number. Second, it might be that the tasks used in this study measure different aspects of information processing which operate in a rather independent manner (Brown et al., 2014). Most importantly, while the stimulus material was similar in the AAT and dot-probe task (i.e., general positive or negative pictures), the IAT was based on a comparison between flowers and insects. The decision for this approach was grounded on several considerations. For one part, we wished to assess a near-universal association, which was robustly found in healthy samples. Apart from that, a pilot study using stimulus material comparable to the AAT and the dot-probe task yielded poor results. This was the case because participants had considerable difficulties in incompatible trials due to a large semantic overlap between attribute words and target stimuli, resulting in extremely large RTs and error percentages. Hence, it might be that convergence between tasks was undermined because of different stimuli used, which in turn led to different processes measured. From this point of view, future studies should pay special attention to stimulus content when comparing different indirect tasks. In the present case, higher across-tasks correlations could have been expected if stimulus content would have been as similar as possible (i.e., pictures of insects and flowers throughout all three tasks).

Furthermore, bias scores were not consistently associated with self-report measures, undermining the validity of the tasks used here. Even though we did not expect a strong correlation between implicit and explicit measures, as the presumed benefit of using indirect measures is that they are thought to capture processes that lie outside of conscious awareness, a pure lack of association is still striking. The same methodological (i.e., low reliability and power) and conceptual reasons (i.e., correspondence between measures) may account for the lack of association between cognitive biases and self-report as those for the lack of convergence between biases. In a recent commentary, Dang et al. (2020) emphasize the fact that behavioral and self-report tasks require different response processes and therefore might be weakly correlated. For instance, while behavioral measures rely on performance such as reaction times, self-report tasks are based on reflections of performance. Furthermore, items used in self-report measures are often more specific than general performance differences (i.e., general information processing biases), leading to a mismatch in the level of detail. These issues contribute to rather low implicit–explicit correlations to begin with and, as outlined above, our power was insufficient to detect small effects. As this difficulty is frequently encountered in correlational designs and associated research, some recent work dealt with the question of how to increase power through sophisticated methodological approaches. For instance, Toffalini et al. (2021) have demonstrated that the use of repeated rather than single measurements (i.e., three times before treatment and three times after treatment) has merits in increasing power. Although their approach is not entirely applicable to our data due to differences in study design and data collection, an exploratory analyses based on aggregated bias scores across sessions provides some indications that implicit–explicit correlation can be improved when scores are summed across sessions (see supplemental material appendix). Hence, a promising approach in future studies might lie in collecting RT data on several time points in close temporal stability, especially in contexts where associations are expected to be low, yet meaningful.

Finally, it might be that convergence across different cognitive biases and/or correlations between implicit and explicit measures are more pronounced in psychopathology than in psychological well-being and functioning. Thus, stronger interrelations might be observed in clinical samples than in subclinical or healthy populations (Bar-Haim et al., 2007). Hence, our findings warrant replication in clinical samples, including anxious or depressed patients or individuals suffering from substance use disorders.

### Implications

In light of the continually expanding research interest in cognitive biases, the development of ever more widespread research questions, and a steady shift from basic mechanisms-focused to applied clinical research (Blackwell et al., 2017), it becomes more and more important to match task requirements with the exact research question asked. For example, in studies aiming at examining group differences (i.e., clinical vs. healthy groups), information-processing tasks should be highly reliable in terms of internal consistency. On the other hand, if researchers are interested in the effects of therapy on cognitive biases, the task should be reliable in terms of temporal stability in order to ensure that changes in cognitive biases are attributed to the therapy instead of random fluctuations over time. Finally, when the main endeavor would be to reduce cognitive biases in order to contribute to symptom reduction, this would require the task to be valid (i.e., correlated with self-reported or observed symptoms). Therefore, tasks should be chosen which are appropriate to the specific research question in mind.

### Conclusions

Recent years have witnessed a positive development in improving research practice in psychology. So far, this includes preregistered analyses, open-access data, a priori power calculations, and the integration of both frequentist and Bayesian analyses. However, the question whether our frequently used experimental paradigms hold adequate psychometric characteristics has received little attention. Even though some researchers have addressed this question systematically (Ataya et al., 2012; Brown et al., 2014; Hedge et al., 2018), most studies fail to report reliability estimates of cognitive bias tasks on a standard basis. The present study aimed at filling this gap by applying multiple time points and assessment devices to investigate psychometric properties of three frequently used tasks in experimental psychopathology. We found that reliability estimates varied largely across tasks, time points, and measurement devices. Additionally, there was little evidence for the validity of tasks, as convergence across bias scores and associations with self-report measures were low. Although our results remain preliminary, given the limited sample size, and while our findings warrant further replication (especially in a clinical sample), several conclusions can be drawn that we have summarized in Table 5. First, research should adopt a routine practice to estimate and report psychometric properties of experimental paradigms. Although this notion applies to all fields of psychological research, this is particularly important in cognitive bias research where the tasks used are continuously modified depending on the specific research question (i.e., stimuli type, trial number, instructions used, etc.). As such, results drawn from studies that do not report psychometric properties or report poor reliability should be interpreted with caution. Second, we appreciate research that focuses on developing more reliable assessment tools (MacLeod & Grafton, 2016; Price et al., 2015; Rodebaugh et al., 2016), including searching for optimal ways to calculate bias scores and improve measurement accuracy (i.e., through eye tracking techniques or more accurate measurement devices such as response pads). Finally, we encourage researchers to choose tasks that fit their research question (i.e., focus on individual differences, experimental research, or clinical trials aimed at capturing change over time). For example, some tasks might be better suited for correlational analyses (i.e., those with good reliability), while others might perform better in experimental research (i.e., tasks with moderate temporal stability and/or high sensitivity to change) or in training studies as a means for bias modification (i.e., tasks that correlate with symptoms or behavior).

**Table 5:** General recommendations for standard practices in cognitive bias research

**Recommendations for the planning stage**	
- Select experimental tasks and bias indices that have proven reliable in previous research - Use adequate trial number per condition (i.e., n = 100) - Use tasks that are most suitable to answer the precise research question asked (i.e., in interventional research, tasks should be sensitive to change, in experimental research tasks should be internally consistent) - In training studies (Cognitive Bias Modification), decide which tasks to use for bias assessment and bias modification - Use adequate hardware and software that was particularly designed to accurately measure reaction times in milliseconds (i.e., response pads are favorable over keyboards)	
**Recommendations for analyses**	
- Use computational algorithms (i.e., calculations for bias scores) that have been previously used and for which psychometric properties have been reported and established - Test adaptations to the computational algorithm in an effort to increase psychometric quality of the task - Calculate internal consistency for each individual measurement and version of the task - If more than one measurement with the same task was conducted, calculate test-retest reliability	
**Recommendations for reporting**	
- Always report psychometric characteristics of each experimental task and measurement point - Specify the reliability estimation method used - Report the software method used and the analytic pathway undertaken to reach a reliability estimate - For split-half reliabilities, report both the uncorrected estimate and the Spearman-Brown-corrected estimate, where applicable - Facilitate transparency and comparability of reliability estimates by reporting the complete analysis procedure (i.e., error treatment, outlier rejection) and/or provide the analysis code - Provide open data to enable other researchers to perform additional tests	

## Supplementary Information

ESM 1 (PDF 1138 kb)

## Footnotes

1 Please note that a Spearman-Brown correction was not used when reporting consistency results based on the IAT D-score. The reason for this is that a split-half reliability with Spearman-Brown correction is typically used in cases where a sum score is calculated from a set of items, and the item sets have half the number of responses. However, as the IAT is scored by averaging two D-scores, each of which contains information from many RTs, the Spearman-Brown correction is not appropriate in this context.
