# Supplementary material for: Same same, but different: A psychometric examination of three frequently used experimental tasks for cognitive bias assessment in a sample of healthy young adults
Source: Behav Res Methods. 2022 Jun 1;55(3):1332–51. doi: 10.3758/s13428-022-01804-9 (PMC10126031; doi:10.3758/s13428-022-01804-9)
Supplement: Supplementary file 1 — (PDF 1138 kb) [file 13428_2022_1804_MOESM1_ESM.pdf]

Supplemental material appendix

Here, we report additional detailed results on reliability and validity analyses that were not included into the main results of the manuscript due to reasons of space or the explorative nature of the analyses. These extended results include i) confidence intervals around the r estimate for reliability and validity analyses; ii) psychometric properties for mean reaction times (i.e., mean RTs for compatible blocks; mean RTs for incompatible blocks) as opposed to difference scores; iii) the influence of different scoring algorithms for bias score calculation (d-score vs. difference scores) on validity (i.e., comparability across tasks); and iv) an investigation of the use of repeated measurements (i.e., aggregating bias scores over sessions) for reliability and implicit-explicit correlations.

I. Correlations complemented by Confidence Intervals

Table A1

Internal consistency (split-half correlation) for cognitive bias assessment tasks

**Task**	**Device**				
AAT		*Time*	*r*	r_{SB}	*n*
**Negative Approach Bias**	PC	1	0.55*** [.36;.69]	0.71*** [.61;.83]	74
		2	0.60*** [.43;.74]	0.75*** [.62;.84]	69
		3	0.45** [.24;.62]	0.62*** [.45;.674]	73
		4	0.56*** [.38; .71]	0.72*** [.58;.82]	70
		5	0.60*** [.43;.734	0.75*** [.63;.84]	70
**Positive Approach Bias**		1	0.43*** [.22;.60]	0.60*** [.43;.73]	74
		2	0.63*** [.45;.75]	0.77*** [.65;.85]	69
		3	0.63*** [.46;.75]	0.77*** [.66;.85]	73
		4	0.58*** [.40; .72]	0.73*** [.60;.82]	70
		5	0.56*** [.37;.71]	0.72*** [.58;.82]	70
**Negative Approach Bias**	touch-screen	1	0.48*** [.28;.64]	0.65*** [.50;.76]	74
		2	-0.12 [-.36;.14]	-0.21 [-.43;.03]	69
		3	0.35** [.11;.55]	0.52*** [.32;.67]	70
		4	0.58*** [.40;.72]	0.73*** [.60;.82]	73
		5	0.58*** [.38;.73]	0.73*** [.60;.82]	69
**Positive Approach Bias**		1	0.29* [.07;.49]	0.45*** [.25;.62]	74
		2	0.58*** [.37;.74]	0.73*** [.60;.82]	69
		3	0.57*** [.36;.72]	0.73*** [.60;.82]	70
		4	0.08 [-.15;.30]	0.15 [-.08;.37]	73
		5	0.18 [-.08;.42]	0.31** [.08;.51]	69
Dot probe task					
**Attentional Bias**	PC	1	-0.18 [-.39; .04]	-0.31** [-.50;-.09]	76
		2	0.16 [-.09;.38]	0.28* [.04;.49]	67
		3	0.08 [-.16;.31]	0.15 [-.09;.37]	71
		4	0.03 [-.20;.26]	0.06 [-.17;.29]	72
		5	-0.30* [-.50;-.07]	-0.46*** [-.63;-.25]	71

**Attentional Bias**	touch-screen	1	-0.04 [-.28;.20]	-0.08 [-.31;.16]	68
		2	-0.11 [-.34;.13]	-0.20 [-.42;.05]	65
		3	-0.08 [-.31;.15]	-0.15 [-.37;.09]	73
		4	0.01 [-.22;.24]	0.02 [-.21;.25]	72
		5	0.10 [-.14;.32]	0.18 [-.06;.40]	67
IAT					
**Association Bias (d-score)**	PC	1	0.16 [-.11;.41]	-	55
		2	0.24^{*} [.004;.46]	-	68
		3	0.42^{***} [.21;.60]	-	70
		4	0.51^{***} [.31;.66]	-	72
		5	0.48^{***} [.27;.64]	-	71
**Association Bias (d-score)**	touch-screen	1	0.60^{***} [.43;.73]	-	75
		2	0.37^{**} [.14;.56]	-	65
		3	0.50^{***} [.30;.65]	-	73
		4	0.36^{**} [.13;.54]	-	72
		5	0.38^{**} [.16;.57]	-	69

Note. All bias scores were measured at five different timepoints. AAT: Approach-Avoidance Task; IAT: Implicit Association Task; *r*: Pearson's correlation coefficient; r_{SB}: Spearman-Brown correction; 1: Timepoint 1; 2: Timepoint 2; 3: Timepoint 3; 4: Timepoint 4; 5: Timepoint 5; * p < .05; ** p < .01; *** p < .001; n: number of observed cases for each task; "r" denotes the correlation between the odd and even trials (in the case of AAT and dot probe) or the correlation between the first (practice) and second (test) block (in the case of the IAT), respectively, and is based on multiple imputation. Due to the fact that internal consistency for the IAT was based in blocks rather than a set of items, a Spearman-Brown correction was not applicable to this case. 95%-Confidence Intervals are given in square brackets.

Table A2

Temporal stability (test-retest reliability) for cognitive bias assessment tasks

**Task**	**Device**	**Test-retest correlations (r)**				
		Time				
AAT			*T1*	*T2*	*T3*	*T4*
**Negative Approach Bias**	PC	*T2*	0.44*** [.23;.61]			
		T3	0.45*** [.24;.61]	0.54*** [.35;.69]		
		T4	0.47*** [.26;.63]	0.48*** [.28;.64]	0.38*** [.16;.57]	
		T5	0.43*** [.22;.60]	0.41*** [.20;.59]	0.65*** [.49;.77]	0.54*** [.35;.67]
**Positive Approach Bias**		*T2*	0.33** [.09;.53]			
		T3	0.25* [.02;.46]	0.62*** [.45;.75]		
		T4	0.34** [.11;.54]	0.42*** [.21;.60]	0.38*** [.16;.56]	
		T5	0.34** [.11;.54]	0.41*** [.19;.59]	0.62*** [.45;.75]	0.49*** [.29;.65]
**Negative Approach Bias**	Touchscreen	*T2*	-0.05 [-.28;18]			
		T3	0.00 [-.23;.23]	0.21 [-.05;.44]		
		T4	0.22 [-.01;.43]	-0.02 [-.26;.22]	0.32** [.09;.53]	
		T5	0.03 [-.20;.26]	0.22 [-.02;.43]	0.24 [-.01;.46]	0.29* [.06;.49]
**Positive Approach Bias**		*T2*	0.07 [-.16;.29]			
		T3	0.25* [.02;.46]	0.00 [-.24;.25]		
		T4	0.17 [-.06;.38]	0.15 [-.15;.42]	0.17 [-.07;.39]	
		T5	0.12 [-.11;.34]	-0.07 [-.30;.17]	0.29* [.05;.50]	-0.19 [-.41;.05]
Dot probe task						
**Attentional Bias**	PC	*T2*	-0.14 [.37;.10]			
		T3	-0.20 [-.41;.03]	-0.19 [-.41;.04]		
		T4	-0.13 [-.35;.11]	0.08 [-.16;.31]	0.10 [-.13;.33]	
		T5	0.09 [-.15;.31]	0.20 [-.08;.45]	0.07 [-.17;.30]	0.12 [-.11;.35]
**Attentional Bias**	Touchscreen	*T2*	0.00 [-.26;.25]			
		T3	0.04 [-.20;.27]	-0.08 [-.32;.17]		
		T4	0.15 [-.09;.38]	0.10 [-.16;.34]	0.02 [-.21;.25]	
		T5	0.03 [-.22;.27]	0.25* [.001;.48]	-0.09 [-.32;.15]	0.01 [-.23;.25]
IAT						
**Association Bias (d-score)**	PC	*T2*	0.36** [.10;.57]			
		T3	0.50*** [.27;.69]	0.35** [.13;.55]		
		T4	0.44*** [.20;.63]	0.38** [.16;.57]	0.58*** [.41;.72]	
		T5	0.36** [.09;.58]	0.40*** [.17;.59]	0.26* [.04;.47]	0.31*** [.09;.51]
**Association Bias (d-score)**	Touchscreen	*T2*	0.54*** [.35;.70]			
		T3	0.49*** [.29;.65]	0.39** [.16;.57]		
		T4	0.31** [.09;.51]	0.36** [.13;.56]	0.51*** [.32;.66]	
		T5	0.17 [-.06;.39]	0.25* [.01;.47]	0.13 [-.11;.35]	0.22 [-.02;.44]

Note. AAT: Approach-Avoidance Task; IAT: Implicit Association Task; RT: reaction time; r: Pearson's correlation co-efficient; T1: Timepoint 1; T2: Timepoint 2; T3: Timepoint 3; T4: Timepoint 4; T5: Timepoint 5; * p < .05; ** p < .01; *** p < .001. 95%-Confidence Intervals are given in square brackets.

Table A3

Pearson's correlations (r) between cognitive biases of the PC and touchscreen assessments at the first measurement time point with self-reported trait variables

	PC assessment				Touchscreen assessment			
**Variable**	Negative AAT Bias	Positive AAT Bias	ATT Bias	IAT Bias	Negative AAT Bias	Positive AAT Bias	ATT Bias	IAT Bias
**NEO-FFI**								
Openness	-0.07[-.29;.16]	0.08[-.16;.31]	-0.18[-.39;.06]	-0.18[-.43;.08]	0.11[-.13;.34]	-0.13[-.35;.10]	-0.03[-.27;.20]	-0.01[-.24;.22]
Conscientiousness	0.01[-.22;.24]	0.21[-.03;.43]	0.02[-.20;.25]	**0.38**[.11;.59]**	0.09[-.14;.31]	0.11[-.12;.33]	-0.05[-.28;.17]	**0.40***[.19;.58]**
Extraversion	-0.08[-.31;.15]	0.01[-.23;.25]	-0.06[-.29;.17]	0.17[-.09;.41]	0.04[-.19;.26]	0.10[-.13;.32]	-0.01[-.25;.23]	0.06[-.17;.29]
Agreeableness	-0.10[-.32;.13]	0.02[-.22;.25]	-0.14[-.36;.09]	0.20[-.08;.45]	0.02[-.21;.24]	-0.06[-.28;.17]	0.02[-.22;.26]	0.04[-.19;.27]
Neuroticism	-0.06[-.29;.17]	0.00[-.23;.23]	-0.03[-.25;.20]	-0.09[-.34;.17]	-0.07[-.29;.17]	-0.09[-.31;.14]	0.03[-.22;.28]	0.04[-.19;.26]
**ANPS**								
SEEKING	0.02[-.21;.24]	0.13[-.11;.35]	-0.04[-.27;.19]	-0.01[-.26;.24]	0.06[-.17;.29]	-0.01[-.24;.22]	-0.15[-.37;.09]	0.06[-.18;.28]
PLAY	-0.15[-.36;.09]	-0.09[-.32;.15]	-0.04[-.27;.19]	0.17[-.09;.40]	0.08[-.15;.30]	0.00[-.23;.22]	-0.01[-.24;.23]	0.07[-.16;.29]
CARE	**-0.25*[-.46;.03]**	-0.07[-.30;.17]	-0.10[-.32;.13]	0.01[-.24;.27]	-0.06[-.29;.18]	-0.01[-.23;.22]	-0.05[-.28;.19]	0.05[-.18;.28]
FEAR	0.09[-.15;.31]	0.04 [-.20;.28]	-0.05[-.28;.18]	-0.09[-.35;.18]	0.01[-.22;.24]	0.00[-.23;.23]	-0.09[-.32;.16]	0.01[-.22;.24]
ANGER	0.07[-.16;.30]	0.08[-.15;.31]	0.07[-.16;.30]	0.08[-.21;.36]	0.03[-.20;.26]	-0.02 [-.25;.20]	0.02[-.23;.26]	0.09[-.14;.31]
SADNESS	0.05[-.18;.09]	0.13[-.12;.36]	-0.08[-.31;.15]	-0.04[-.30;.22]	0.07[-.17;.30]	-0.17[-.38;.06]	0.07[-.18;.31]	0.00[-.23;.23]
SPIRITUALITY	-0.13[-.35;.10]	-0.06[-.30;.18]	-0.19[-.40;.04]	-0.04[-.30;.22]	0.07[-.16;.29]	-0.03[-.25;.20]	0.02[-.22;.26]	-0.09[-.32;.14]
LIE	-0.01[-.24;.22]	0.01[-.23;.24]	0.09[-.14;.31]	0.11[-.15;.36]	-0.05[-.27;.18]	0.10[-.13;.32]	0.00[-.24;.23]	0.08[-.16;.31]
**Positive mental health (PMH)**	0.06[-.17;.28]	0.05[-.19;.29]	-0.02[-.25;.21]	0.12[-.13;.37]	0.17[-.06;.39]	0.16[-.17;.37]	-0.08[-.33;.18]	0.09[-.14;.32]
**TRAIT ANXIETY**								
STAI-G	0.15[-.08;.36]	0.01[-.22;.24]	0.02[-.21;.25]	-0.20[-.43;.06]	-0.18[-.40;.05]	-0.06[-.28;.17]	0.02[-.23;.26]	-0.18[-.40;.05]

ASI-4	0.00[-.23;.23]	-0.12[-.34;.12]	-0.11[-.34;.13]	-0.06[-.33;.21]	-0.09[-.31;.15]	0.10[-.14;.32]	-0.08[-.31;.16]	0.08[-.15;.30]
ABI: Vigilance	-0.03[-.26;.20]	-0.15[-.37;.09]	0.16[-.07;.38]	0.07[-.19;.33]	0.01[-.22;.24]	**0.28*[.06;.47]**	-0.18[-.40;.07]	-0.05[-.27;.19]
ABI: Cognitive Avoidance	0.10[-.14;.32]	0.19[-.05;.41]	0.17[-.06;.39]	0.08[-.19;.33]	-0.04[-.27;.19]	0.19[-.14;.32]	-0.07[-.31;.17]	0.07[-.15;.30]
**Sensation seeking**								
NISS: Need for Stimulation	-0.03[-.26;.20]	-0.08[-.31;.17]	-0.02[-.25;.21]	-0.08[-.34;.19]	-0.07[-.29;.16]	0.17[-.06;.39]	-0.11[-.34;.13]	-0.05[-.28;.18]
NISS: Avoidance of Rest	-0.05[-.28;.18]	0.03[-.21;.27]	-0.14[-.36;.09]	0.17[-.09;.41]	0.02[-.21;.25]	0.13[-.10;.35]	-0.06[-.27;.18]	-0.06[-.28;.17]
NISS: Sum Score	-0.05[-.28;.19]	-0.04[-.28;.20]	-0.09[-.31;.14]	0.02[-.24;.29]	-0.04[-.27;.19]	0.20[-.03;.40]	-0.11[-.34;.12]	-0.07[-.30;.16]
**Disgust Scale**	0.12[-.12;.34]	0.14[-.10;.37]	-0.07[-.30;.18]	0.01[-.26;.28]	0.00[-.24;.23]	0.03[-.20;.26]	0.03[-.23;.27]	0.14[-.09;.37]

Note. NEO-FFI: Neuroticism-Extraversion-Openness-Five-Factor-Inventory; ANPS: Affective Personality Scales; STAI-G: State-Trait-Anxiety-Inventory German version; ASI-4: Anxiety Sensitivity Index 4; ABI: Anxiety Coping Index; NISS: Need for Sensation Seeking; AAT = Approach/Avoidance Bias derived from the Approach-Avoidance Task; ATT Bias = Attentional Bias derived from the dot probe task; IAT Bias = Association Bias (D-Score) derived from the Implicit Association Test; *p < .05, **p < .01, ***p < .001; significant correlations are shown in bold; 95% Confidence Intervals are given in square brackets.

II. Psychometric properties of mean reaction times

Internal consistency: Split-half correlations

Next to investigating internal consistency for bias scores derived from the AAT, the dot probe task and the IAT (main manuscript), here we analyze reliability for mean reaction times (RTs) per condition (i.e., mean RTs for compatible or incompatible condition). Internal consistency of the measurements were quantified using the split-half-method. More precisely, reliability estimates for mean RTs derived from the AAT and the dot probe task were determined by means of correlations between the odd and even trial numbers respectively. Internal consistency from the IAT was calculated by correlating the first (practice) and the second (test) block as recommended by Greenwald and colleagues (2003). Detailed results are displayed in Table A4.

AAT. Overall, split-half reliability coefficients demonstrated good to excellent internal consistency for mean condition RTs (both PC- and touchscreen versions) and ranged between r = .64 (r_{SB} = .78) and r = .94 (r_{SB} = .97).

Dot-probe task. Split-half correlations for mean RTs were high for both versions of the task and ranged between r = .71 (r_{SB} = .83) and r = .90 (r_{SB} = .95).

IAT. Split-half correlations were acceptable for mean condition RTs and ranged between r = .30 and r = .86. In general, reliability was somewhat greater for the touchscreen version of the task than for the PC-version.

Temporal stability: Test-retest reliability

Stability across time of the respective mean RTs was inferred from their bivariate autocorrelations. Detailed results are displayed in Table A5.

AAT. Mean RTs exhibited good test-retest correlations when assessed via PC. Estimates were lower, but still substantial for touchscreen-based assessment.

Dot-probe Task. Estimates of test-retest reliability were high for condition RTs (all but two coefficients > .50).

IAT. Autocorrelations of mean RTs were acceptable with most coefficients >.50.

Overall, all three tasks showed moderate to good internal consistency and temporal stability when mean RTs were analyzed (Koo & Li, 2016; Schmukle et al., 2005). Compared to the findings in the main manuscript, split-half and test-retest correlations for mean RTs yielded high consistencies and were in general larger than reliability estimates obtained from bias scores. It should be mentioned in this context, however, that reliability estimates for mean RTs would always turn out to be somewhat higher than those for difference scores. This is due to the fact that measurement error from the two trials/blocks (i.e., compatible vs. incompatible trials) is compounded when combined into a single index, resulting in an attenuation of correlation coefficients (Overall

& Woodward, 1975; see also Brown et al., 2014; Enkavi et al., 2019). Furthermore, mean condition RTs are difficult to interpret, as they are limited in capturing individual differences in information processing preferences. High correlations within and across sessions might also highlight general response speed, independent of emotional condition. As bias scores are a better index of information processing preferences than mere mean RTs, the herein presented results should be interpreted with caution and interested researchers should abstain from relying on mean RTs only when interpreting psychometric properties of behavioral RT-based tasks.

Table A4

Internal consistency (split-half correlation) and descriptives for cognitive bias assessment tasks

**Task**	**Device**					**1. Half**		**2. Half**	
AAT		*Time*	*r*	r_{SB}	*n*	*M*	*SD*	*M*	*SD*
Negative Push RT	PC	1	.91***	.95***	74	769	131	765	121
		2	.89***	.94***	69	676	113	673	100
		3	.88***	.94***	73	654	94	647	77
		4	.87***	.93***	70	632	77	628	78
		5	.88***	.94***	70	623	82	616	85
Negative Pull RT		1	.93***	.96***	74	764	148	777	150
		2	.93***	.96***	69	672	118	676	135
		3	.91***	.95***	73	639	100	644	95
		4	.88***	.94***	70	625	98	628	92
		5	.90***	.95***	70	626	87	626	85
Positive Push RT		1	.89***	.94***	74	733	103	753	116
		2	.88***	.94***	69	665	96	676	112
		3	.90***	.95***	73	644	86	651	84
		4	.91***	.95***	70	621	73	628	789
		5	.91***	.95***	70	615	87	616	73
Positive Pull RT		1	.87***	.93***	74	747	146	747	129
		2	.93***	.96***	69	658	112	667	116
		3	.93***	.96***	73	634	92	637	92
		4	.86***	.92***	70	616	88	622	79
		5	.92***	.96***	70	615	77	618	81
Negative Push RT	touch-screen	1	.82***	.90***	74	972	398	997	414
		2	.72***	.84***	69	868	370	857	332
		3	.83***	.91***	70	800	271	781	260
		4	.86***	.92***	73	731	190	731	195
		5	.89***	.94***	69	709	167	709	157
Negative Pull RT		1	.84***	.91***	74	976	440	1002	419
		2	.77***	.87***	69	870	367	878	435
		3	.84***	.91***	70	773	244	777	248
		4	.94***	.97***	73	739	277	736	259
		5	.86***	.92***	69	702	212	698	182

Positive Push RT		1	.73***	.84***	74	935	364	966	408
		2	.71***	.83***	69	840	314	870	392
		3	.90***	.95***	70	767	225	779	245
		4	.92***	.96***	73	733	205	733	201
		5	.64***	.78***	69	692	141	718	241
Positive Pull RT		1	.84***	.91***	74	931	328	954	380
		2	.88***	.94***	69	97	566	888	615
		3	.84***	.91***	70	780	252	791	307
		4	.83***	.91***	73	724	230	729	238
		5	.83***	.91***	69	692	172	686	151
Dot probe task									
Incompatible RT	PC	1	.85***	.92***	76	367	55	359	53
		2	.84***	.91***	67	338	45	344	53
		3	.88***	.94***	71	341	49	342	59
		4	.74***	.85***	72	336	58	326	43
		5	.77***	.87***	71	341	49	333	44
Compatible RT		1	.82***	.90***	76	358	49	358	61
		2	.80***	.89***	67	339	50	336	45
		3	.81***	.90***	71	337	57	340	57
		4	.77***	.87***	72	330	49	327	53
		5	.71***	.83***	71	334	48	336	52
Incompatible RT	touch-screen	1	.90***	.95***	68	429	69	427	71
		2	.79***	.88***	65	386	56	385	54
		3	.86***	.92***	73	376	51	379	56
		4	.81***	.90***	72	373	48	376	48
		5	.76***	.86***	67	377	45	377	48
Compatible RT		1	.88***	.94***	68	426	66	421	71
		2	.87***	.93***	65	382	59	384	60
		3	.86***	.92***	73	378	57	377	56
		4	.88***	.94***	72	371	46	367	47
		5	.81***	.90***	67	374	51	375	44
IAT									
Incompatible RT	PC	1	.31*	-	55	937	234	915	328
		2	.71***	-	68	821	222	799	200
		3	.69***	-	70	789	198	785	181
		4	.72***	-	72	723	163	767	199
		5	.66***	-	71	749	22	76	213
Compatible RT		1	.30*	-	55	697	156	652	122
		2	.66***	-	68	633	130	649	204
		3	.59***	-	70	618	134	620	132
		4	.77***	-	72	638	150	647	193
		5	.49***	-	71	663	263	633	162

Incompatible RT	touch-screen	1	.77***	-	75	1021	241	946	208
		2	.86***	-	65	900	317	831	269
		3	.57***	-	73	830	284	844	319
		4	.73***	-	72	791	220	802	218
		5	.67***	-	69	822	285	825	218
Compatible RT		1	.73***	-	75	789	162	754	170
		2	.65***	-	65	714	188	714	344
		3	.61***	-	73	690	168	688	183
		4	.72***	-	72	697	195	706	188
		5	.79***	-	69	713	182	683	159

Note. Reaction times (RTs) were measured at five different timepoints. AAT: Approach-Avoidance Task; IAT: Implicit Association Task; *r*: Pearson's correlation coefficient; r_{SB}: Spearman-Brown correction; 1: Timepoint 1; 2: Timepoint 2; 3: Timepoint 3; 4: Timepoint 4; 5: Timepoint 5; * $p < .05$; ** $p < .01$; *** $p < .001$; M: mean RT; SD: standard deviation; n: number of observed cases for each task; "r" denotes the correlation between the odd and even trials (in the case of AAT and dot probe) or the correlation between the first (practice) and second (test) block (in the case of the IAT) respectively and is based on multiple imputation. Due to the fact that internal consistency for the IAT was based in blocks rather than a set of items, a Spearman-Brown correction was not applicable to this case.

Table A5

Temporal stability (test-retest reliability) for cognitive bias assessment tasks

**Task**	**Device**		**Test-retest correlations (r)**			
			Time			
AAT			*T1*	*T2*	*T3*	*T4*
Negative Push RT	PC	*T2*	.81***			
		T3	.76***	.82***		
		T4	.68***	.74***	.77***	
		T5	.68***	.76***	.79***	.83***
Negative Pull RT		*T2*	.76***			
		T3	.71***	.79***		
		T4	.67***	.68***	.72***	
		T5	.70***	.68***	.75***	.82***
Positive Push RT		*T2*	.81***			
		T3	.75***	.82***		
		T4	.68***	.72***	.80***	
		T5	.69***	.75***	.82***	.82***
Positive Pull RT		*T2*	.74***			
		T3	.65***	.78***		
		T4	.67***	.70***	.71***	
		T5	.70***	.71***	.76***	.79***
Negative Push RT	Touchscreen	*T2*	-.27*			
		T3	.23*	.38**		
		T4	.36**	.52***	.35**	
		T5	.43***	.55***	.40***	.74***
Negative Pull RT		*T2*	.24*			
		T3	.13	.40**		
		T4	.32**	.39***	.35**	
		T5	.32**	.43***	.39***	.54***
Positive Push RT		*T2*	.30**			
		T3	.25*	.39***		
		T4	.26*	.48***	.33**	
		T5	.41***	.52***	.44***	.69***
Positive Pull RT		*T2*	.35**			
		T3	.29*	.42***		
		T4	.37**	.58***	.34**	
		T5	.28*	.44***	.43***	.60***
Dot probe task						

Incompatible RT	PC	*T2*	.55***			
		T3	.47***	.70***		
		T4	.56***	.72***	.71***	
		T5	.57***	.71***	.74***	.72***
Compatible RT		*T2*	.63***			
		T3	.52***	.66***		
		T4	.54***	.60***	.64***	
		T5	.59**	.71***	.68***	.63***
Incompatible RT	Touchscreen	*T2*	.54***			
		T3	.67***	.81***		
		T4	.45***	.52***	.70***	
		T5	.64***	.62***	.71***	.67***
Compatible RT		*T2*	.56***			
		T3	.58***	.86***		
		T4	.42***	.55***	.70***	
		T5	.58**	.63***	.71***	.65***
IAT						
Incompatible RT	PC	*T2*	.65***			
		T3	.64***	.72***		
		T4	.63***	.72***	.71***	
		T5	.49***	.62***	.52***	.72***
Compatible RT		*T2*	.50***			
		T3	.63***	.60***		
		T4	.36**	.62***	.64***	
		T5	.41**	.65***	.52***	.48***
Incompatible RT	Touchscreen	*T2*	.47***			
		T3	.53***	.38**		
		T4	.49***	.60***	.57***	
		T5	.48***	.51***	.54***	.68***
Compatible RT		*T2*	.66***			
		T3	.63***	.55***		
		T4	.58***	.72***	.68***	
		T5	.41***	.33**	.45***	.52***

Note. AAT: Approach-Avoidance Task; IAT: Implicit Association Task; RT: reaction time; r: Pearson's correlation co-efficient; T1: Timepoint 1; T2: Timepoint 2; T3: Timepoint 3; T4: Timepoint 4; T5: Timepoint 5; * p < .05; ** p < .01; *** p < .001.

III. The use of different scoring algorithms for bias score calculation

Criterion validity: Convergence between cognitive bias measures

Here, we report additional results on the comparison between different experimental paradigms for cognitive bias assessment (criterion validity). While in the main manuscript, the calculation of bias scores was based on the most conventional approaches from the literature (that is: difference between median reaction times for the AAT and the dot-probe-task, d-score algorithm for the IAT), to aid comparability across tasks, two additional

bias score calculation methods have been applied in what follows. The first additional analyses employed the d-score algorithm on reaction times (RT) obtained from the AAT and dot-probe task based on the formula:

$$(meanRT_{incompatile} - meanRT_{compatible}) / SD_{pooled} \tag{1}$$

and compared the results achieved with the IAT d-scores. The second additional analyses re-calculated IAT bias scores based on the formula:

$$medianRT_{incompatible} - medianRT_{compatible} \tag{2}$$

and compared the results achieved with the approach-avoidance (AAT) and attentional (dot probe) biases which were based on the same calculation method.

As can be seen in Figure A1, results are comparable to those reported in the main manuscript (conventional bias score calculation). In the PC-assessment, approach biases for positive and negative cues were highly correlated at each measurement time point, with correlations ranging between 0.58 and 0.75. Bias scores across different assessment tasks were mostly not correlated (only exception: significant correlation between approach biases for positive cues and the IAT-score at t_1; r = .27). In the touchscreen-based assessment, other than for t_1 (r = .34), approach biases for positive and negative pictures did not correlate. The only between task correlation appeared for the AAT (positive cues) and the dot probe task at t_3 (r = -.25).

As can be seen in Figure A2, results did not change substantially when applying formula (2) (difference between median condition RTs). In the PC-assessment, approach biases for positive and negative cues were highly correlated at each measurement time point, with correlations ranging between 0.58 and 0.84. Bias scores across different assessment tasks were mostly not correlated (only exception: significant negative correlation between approach biases for negative cues and the IAT-score at t_5; r = -.32). In the touchscreen-based assessment, approach biases for positive and negative pictures did not correlate at the first two measurement points, but correlations were high for the remaining time points (range: .62 - .71). Other than in the main manuscript where no significant interrelations between approach, attentional, and association biases appeared, there was a correlation between approach biases for negative cues and attentional biases (r = .22) and IAT biases (r = .26) at t_4.

Criterion validity: Convergence between cognitive bias measures using the d-score algorithm

a)

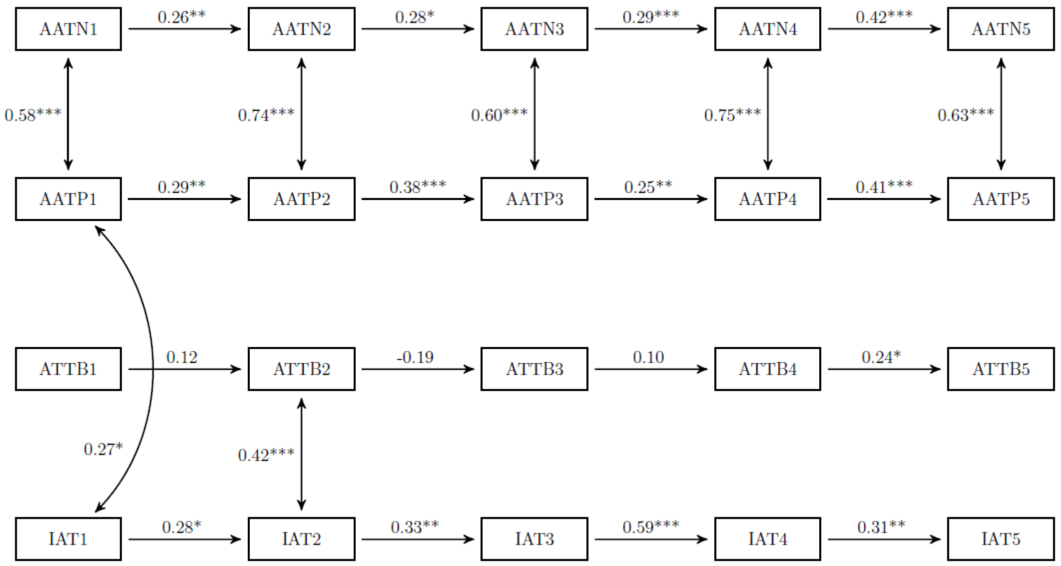

b)

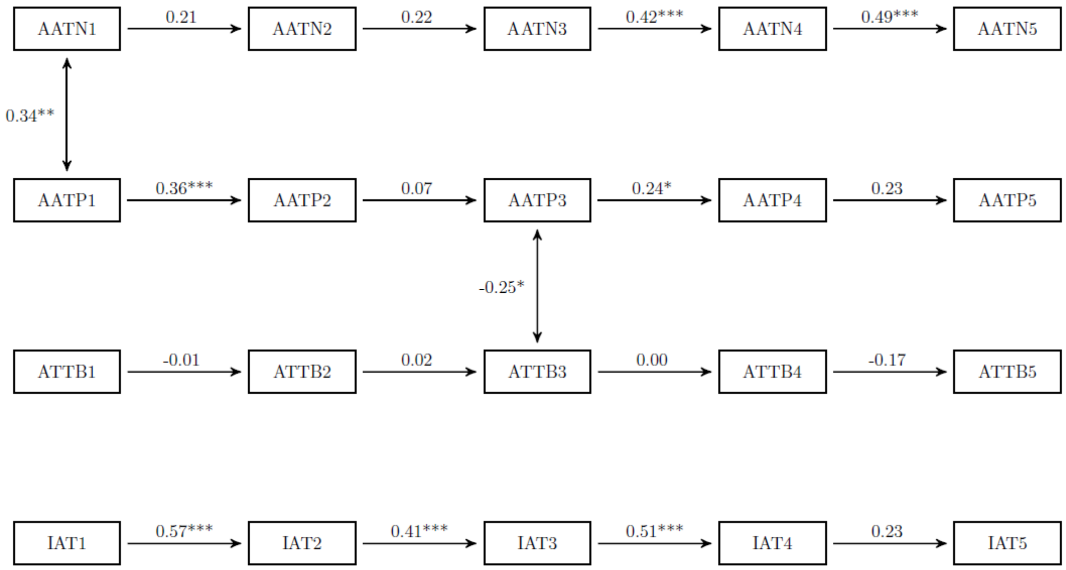

Figure A1. Parallel autoregressive models comparing the stability of the respective biases and their inter-relationships at each measurement timepoint. *Note*. Bias scores were calculated using the d-score algorithm recommended by Greenwald et al. (2003). Panel a) refers to the PC-based assessment; Panel b) to the touchscreen-based-assessment. AATN and AATP denote bias scores towards negative and positive cues respectively, AAT are attentional bias scores; the last row displays IAT bias scores. Numbers 1-5 indicate the respective measurement timepoint. All coefficients are standardized coefficients and were obtained by full information maximum likelihood estimation. All regression coefficients are shown. Only the significant correlations are shown. * p < .05; ** p < .01; *** p < .001.

Criterion validity: Convergence between cognitive bias measures using median difference scores

a)

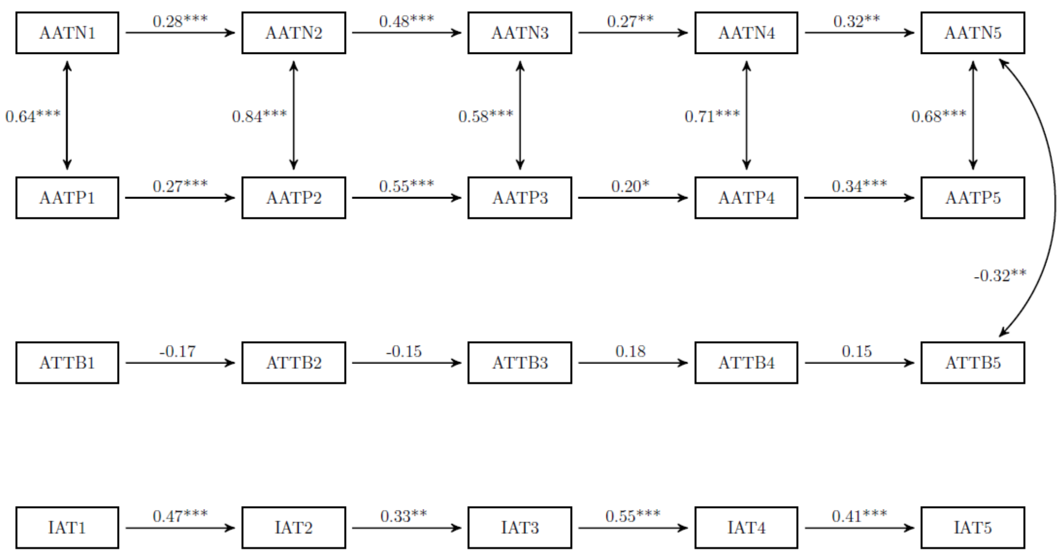

b)

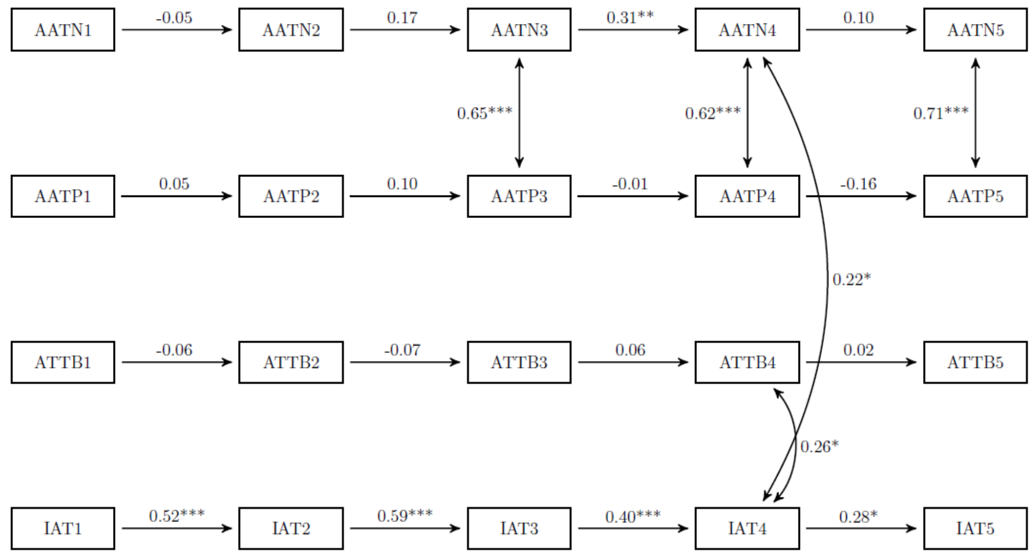

Figure A2. Parallel autoregressive models comparing the stability of the respective biases and their inter-relationships at each measurement timepoint. *Note.* Bias scores were calculated using differences between median condition reaction times. Panel a) refers to the PC-based assessment; Panel b) to the touchscreen-based-assessment. AATN and AATP denote bias scores towards negative and positive cues respectively, AAT are attentional bias scores; the last row displays IAT bias scores. Numbers 1-5 indicate the respective measurement timepoint. All coefficients are standardized coefficients and were obtained by full information maximum likelihood estimation. All regression coefficients are shown. Only the significant correlations are shown. * p < .05; ** p < .01; *** p < .001.

IV. Use of repeated measurements: Aggregating bias scores over measurement time points

Previous work has pointed to the use of combining data from multiple measurements in order to obtain more precise estimates. For instance, Toffalini and colleagues (2021) demonstrated that assessing an outcome multiple times (i.e., three times at pre-treatment and three times at post-treatment) constitutes a feasible way to increase power. Here we report results for aggregated bias scores that were summed over sessions (t_1-t_4). Please note that we decided to use only the first four measurement time points as the fifth time point took place too far apart in time (t_5: +4 weeks vs. t_1-t4: weekly sessions).

Internal consistency: Split-half correlations

Detailed results are depicted in Table A6. As can be seen, the PC-version of the AAT and both versions of the IAT showed good internal consistencies. Reliability for the touchscreen-version of the AAT was somewhat lower, but still substantial. Reliability estimates for the dot probe task failed to reach statistical significance. Results are comparable to those reported in the main manuscript, but internal consistency appears to be somewhat higher when aggregated scores are used.

Table A6

Internal consistency (split-half correlation) and descriptives for aggregated cognitive bias scores

**Task**	**Device**			**2. Half**			
AAT		*r*	r_{SB}	*M*	*SD*	*M*	*SD*
**Approach Bias Negative**	PC	0.71***	0.83***	-2.05	47.25	10.66	56.69
**Approach Bias Positive**		.73***	.84***	4.41	52.9	10.85	50.33
**Approach Bias Negative**	touch-screen	0.24*	0.39***	6.18	136.37	-4.90	115.50
**Approach Bias Positive**		.59***	.74***	-12.04	145.01	1.45	148.09
Dot probe task							
**Attentional Bias**	PC	.015	.03	3.83	14.76	2.52	16.66
**Attentional Bias**	touch-screen	-.11	-.20	2.06	14.79	4.08	15.62
IAT							
**Association Bias**	PC	.63***	-	.54	.33	.51	.29
**Association Bias**	touch-screen	.69***	-	.48	.32	.42	.31

Note. AAT: Approach-Avoidance Task; IAT: Implicit Association Task; r: Pearson's correlation coefficient; r_{SB}: Spearman-Brown correction; 5; * p < .05; ** p < .01; *** p < .001; M: mean bias score; SD: standard deviation; "r" denotes the correlation between the odd and even trials (in the case of AAT and dot probe) or the correlation between the first (practice) and second (test) block (in the case of the IAT) respectively and is based on multiple imputation. Due to the fact that internal consistency for the IAT was based in blocks rather than a set of items, a Spearman-Brown correction was not applicable to this case. Bias scores were aggregated for the first four time points.

Convergence between different assessment devices

Table A7 displays results for correlations between behavioral tasks. As can be seen, approach bias scores for positive and negative cues were strongly correlated, but no correlations appeared between the two different versions of the AAT (PC vs. touchscreen). For the touchscreen-based assessment, approach biases for positive cues were negatively correlated to attentional biases and positively correlated to IAT d-scores. Finally, a strong correlation appeared between the PC- and touchscreen-based assessments of IAT scores. Results are somewhat comparable with those in the main manuscript, but correlations for the two versions of the IAT were stronger when scores were aggregated across measurement time points.

Table A7

Correlations across experimental tasks and assessment devices

	Experimental tasks						
	**AATN (PC)**	**AATP (PC)**	**AATN (touch)**	**AATP (touch)**	**ATT (PC)**	**ATT (touch)**	**IAT (PC)**
**AATP (PC)**	.83***						
**AATN (touch)**	.08	.22					
**AATP (touch)**	.20	.12	.50***				
**ATT (PC)**	.04	.05	-.10	-.07			
**ATT (touch)**	-.10	-.08	-.12	-.26*	.04		
**IAT (PC)**	-.11	.01	-.02	.09	.05	.12	
**IAT (touch)**	-.03	.11	.09	.25*	.13	.08	.72***

Note. AATN and AATP denote approach-avoidance bias scores from the AAT towards negative and positive cues respectively, ATT are attentional bias scores; * p < .05; ** p < .01; *** p < .001.

Construct validity: Association with self-report measures

Correlations between cognitive biases and self-report measures are presented in Table A8. As can be seen, there were only few significant associations between cognitive biases and personality traits or anxiety. Of interest were the positive correlations between IAT-scores and consciousness (for PC: r = .32; for touchscreen: r = .40), the correlations between negative personality traits and approach biases for negative cues as assessed via touchscreen ($r_{neuroticism}$ = .29; r_{FEAR} = .29, r_{ANGER} = .32; $r_{SADNESS}$ = .29), and correlations between positive personality traits and approach and attentional biases as assessed via PC ($r_{AATN;PLAY}$ = -.30; $r_{ATT;CARE}$ = .25). While these relationships are in the expected direction, there were also correlations contrary to the expected direction (negative correlation between approach biases for positive cues and CARE: -.32). Overall, results resemble those reported in the main manuscript. Interestingly, however, somewhat more correlations turned out to be significant if aggregated scores were used as compared to the single (first) measurement (see main manuscript). Still, correlations should be interpreted with caution, given the large number of comparisons.

Taken together, when using combined bias scores (i.e., summed over sessions), results largely resemble those reported in the main manuscript (i.e., per session analysis). However, in some cases, somewhat higher correlations could be reached. In particular, most differences between these exploratory analyses and the findings reported in the manuscript emerged for underpowered analyses. Hence, our findings are in line with Toffalini et al. (2021) and hint to the fact that the use of repeated measurement might increase power, especially in circumstances where power is low. However, a caveat is warranted here: In these exploratory analyses, we aggregated scores across sessions by calculating average scores. Toffalini et al. (2021) on the other hand, suggest using mixed-effects models, with participants as random effects rather than using aggregated scores, since the latter option would lose information on intra-individual variability. This, however, was not possible to perform for the current set of data. In addition, when using repeated measurements as proposed recently, measurement time points should ideally lie in close temporal proximity (i.e., should be performed at the same day) and data should be collected using different versions of the same task.

Table A8

Pearson's correlations (r) between cognitive biases of the PC and touchscreen assessments between aggregated bias scores (t1-t4) and self-reported trait variables

	**PC assessment**				**Touchscreen assessment**			
**Variable**	Negative AAT Bias	Positive AAT Bias	ATT Bias	IAT Bias	Negative AAT Bias	Positive AAT Bias	ATT Bias	IAT Bias
**NEO-FFI**								
Openness	-0.05	0.05	-0.11	-0.10	0.04	-0.02	-0.03	-0.02
Conscientiousness	0.01	0.11	0.11	0.32**	0.15	0.12	0.03	0.40***
Extraversion	-0.14	-0.17	0.08	0.16	-0.17	0.03	0.03	0.08
Agreeableness	-0.17	-0.11	0.23	0.17	-0.11	-0.07	0.19	0.18
Neuroticism	0.03	0.03	0.03	-0.02	0.29*	0.14	0.04	0.03
**ANPS**								
SEEKING	0.10	0.15	0.05	-0.12	0.02	0.00	0.18	0.06
PLAY	-0.30*	-0.32**	0.10	0.03	-0.11	0.01	-0.01	0.01
CARE	-0.15	-0.10	0.25*	0.04	-0.06	0.01	0.04	0.05
FEAR	0.22	0.20	0.07	-0.05	0.29*	0.20	-0.01	0.08
ANGER	0.15	0.14	-0.20	0.05	0.32**	0.12	-0.06	0.06
SADNESS	0.10	0.14	0.08	-0.05	0.29*	0.06	0.09	-0.03
SPIRITUALITY	-0.04	-0.01	0.10	-0.04	0.12	-0.06	0.21	-0.10
LIE	0.07	0.03	0.14	0.06	-0.07	0.00	0.23*	0.03
**Positive mental health (PMH)**	-0.05	-0.07	-0.04	-0.02	-0.15	0.05	0.04	0.04
**TRAIT ANXIETY**								
STAI-G	0.21	-0.03	0.09	-0.15	0.17	0.12	-0.01	-0.11
ASI-4	0.12	0.09	0.04	0.07	0.10	0.17	0.08	0.07

ABI: Vigilance	-0.01	-0.06	0.10	0.06	0.16	0.37***	0.04	0.07
ABI: Cognitive Avoidance	0.08	0.06	0.00	0.08	-0.15	0.15	-0.12	0.06
**Sensation seeking**								
NISS: Need for Stimulation	0.00	-0.03	-0.02	0.04	-0.14	0.07	-0.03	-0.03
NISS: Avoidance of Rest	-0.07	-0.06	0.05	-0.09	0.05	0.14	-0.01	-0.01
NISS: Sum Score	-0.03	-0.05	0.01	-0.01	-0.08	0.13	-0.03	-0.03
**Disgust Scale**	0.21	0.19	0.21	-0.05	0.20	0.14	0.04	0.10

Note. NEO-FFI: Neuroticism-Extraversion-Openness-Five-Factor-Inventory; ANPS: Affective Personality Scales; STAI-G: State-Trait-Anxiety-Inventory German version; ASI-4: Anxiety Sensitivity Index 4; ABI: Anxiety Coping Index; NISS: Need for Sensation Seeking; AAT = Approach/Avoidance Bias derived from the Approach-Avoidance Task; ATT Bias = Attentional Bias derived from the dot probe task; IAT Bias = Association Bias (D-Score) derived from the Implicit Association Test; *p < .05, **p < .01, ***p < .001; Bias scores were aggregated for the first four time points.
